# Insights into the Porretta Terme (northern Apennines, Italy) hydrothermal system revealed by geochemical data on presently discharging thermal waters and paleofluids

**DOI:** 10.1007/s10653-020-00762-5

**Published:** 2020-11-06

**Authors:** Franco Tassi, Paolo S. Garofalo, Filippo Turchetti, Davide De Santis, Francesco Capecchiacci, Orlando Vaselli, Jacopo Cabassi, Stefania Venturi, Stefano Vannini

**Affiliations:** 1 Department of Earth Sciences, University of Florence, Via G. la Pira, 4, 50121 Florence, Italy; 2 Institute of Geosciences and Earth Resources (IGG), National Research Council of Italy (CNR), Via G. La Pira 4, 50121 Firenze, Italy; 3 Department of Biological, Geological and Environmental, University of Bologna, Via Zamboni, 67, 40126 Bologna, Italy; 4 Associazione Fulvio Ciancabilla, Piazza della Libertà, 13, Alto Reno Terme, 40046 Bologna, Italy

**Keywords:** Thermal fluids, Fluid geochemistry, Methane emission, Porretta terme

## Abstract

This study focuses on the geochemical features of the presently discharging thermal and cold springs and on paleofluids from the upstream portion of the Reno river basin (Alto Reno; central–northern Italy). The aim is investigating the primary sources of the modern and fossil fluids and the interactions between deep and shallow aquifers. Paleofluids are from fluid inclusions hosted within euhedral and hopper quartz crystals and consist of a two-phase, liquid–vapor aqueous fluid and a unary CH_4_ fluid. The aqueous inclusions have constant phase ratios and a calculated salinity of ~ 1.5 wt% NaCleq. They homogenize by bubble disappearance at 100–200 °C, whereas the estimated entrapment depth is ~ 3–5.5 km. The paleofluids likely represent the vestiges of the deep and hot, CH_4_-rich, Na^+^–Cl^−^ fluids produced by the interaction between meteoric waters and Triassic and Miocene formations. The modern Na^+^–Cl^−^(HCO_3_^−^) thermal waters originate from meteoric waters infiltrating SW of the study area, at elevation > 800 m a.s.l., circulating within both the Triassic evaporites and the overlying Miocene turbiditic formations, where salt dissolution/precipitation, sulfate reduction, and production of thermogenic CH_4_ occur. The equilibrium temperature of the deep fluid source is ~ 170 °C, corresponding to > 5 km depth. Cold springs are Ca^2+^–HCO_3_^−^ type and show low amounts of biogenic CO_2_ and CH_4_ with no inputs of deep-originated fluids excepting in the immediate surroundings of the thermal area, confirming the lack of significant hydraulic connection between shallow and deep aquifers. We propose a genetic link between the quartz-hosted paleofluid and the thermal waters present in the area.

## Introduction

**Fig. 1:**
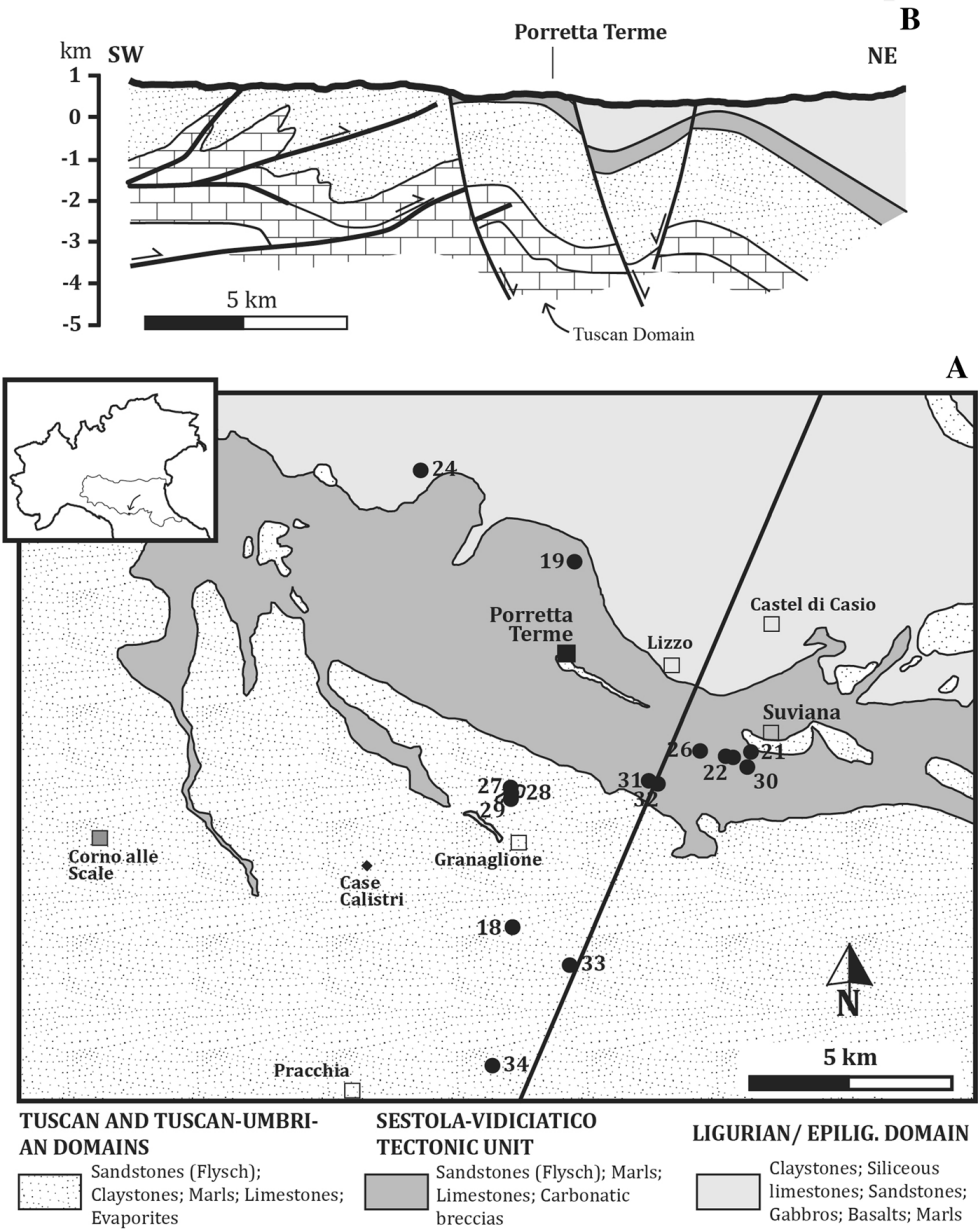
Geological sketch map and cross section of the Porretta Terme area including the main Apennine watershed and the southern portion of the drainage basin of the Reno river (modified from Botti et al. 2017 and Capozzi and Picotti 2010). A. Geological sketch showing the three tectonic–stratigraphic domains that build the area. The contacts between these domains are mainly tectonic and crosscut by normal faults. The numbered symbols mark the water–gas sampling locations of this study. Casa Calistri is a location of quartz fissure veins studied here. B. Geological cross section of the area (marked in A) showing the shallow dipping faults making the fold and thrust belt and the dissecting normal faults (modified from Capozzi and Picotti 2010). Notice that a large part of the Tuscan Domain is buried at depth beneath the area due to horizontal shortening and duplication

The eastern side of the Apennine belt in northern and central Italy hosts a large number and variety of thermal springs. Such springs are frequently highly saline, with a Na^+^–Cl^−^ composition, and are associated with bubbling gases, mostly consisting of CH_4_ with minor H_2_S and light hydrocarbons. Moreover, they often show extrusion of clasts and rock fragments embedded in a clay mineral-rich matrix (e.g., Duchi et al. 2005; Boschetti et al. 2011; Tassi et al. 2012a, b; Cervi et al. 2014). Thermal fluid manifestations and mud volcanism in the external front of the orogenic belt are typically fed by hydrocarbon-rich reservoirs (Mattavelli and Novelli 1987; Minissale et al. 2000; Martinelli and Judd 2004; Capozzi and Picotti 2002, 2010). These reservoirs developed during the Cenozoic as a result of the convergence and subduction of the Adriatic plate beneath the European lithosphere (Boccaletti et al. 1971; Royden et al. 1987; Faccenna et al. 2044). The internal front of the belt is, however, distinct from the external one, as the former hosts brackish springs that are not associated with mud volcanoes. Typical examples of such springs are Bobbio (Boschetti et al. 2011), Miano (Duchi et al. 2005), Vedriano (Bertolini and Gorgoni 2001) and Porretta Terme (Bonoli and Ciancabilla 1995). In particular, the Porretta Terme springs (Fig. 1), which are the object of this study, consist of two groups of waters: (1) Terme Alte springs, located within the Porretta Terme village, showing a Na–Cl composition and outlet temperatures up to 35 °C; (2) Puzzola springs, located close to the Reno river at a distance of ~ 1 km from Terme Alte and discharging Na–Cl waters with a lower salinity than those of the first group, slightly lower outlet temperatures (< 29 °C), and higher contents in S-reduced solutes (Ciancabilla and Bonoli 2010). According to a previous study (Capozzi and Picotti 2010), the Porretta springs are essentially deriving from deep connate waters buried within sediments likely located at > 1000 m depth, where thermogenic CH_4_-rich gases are also produced. The same authors identified the main recharge area in the Tertiary sequence outcropping NW of Porretta. Alternatively, meteoric waters were supposed to infiltrate through the turbidite formation outcropping at Mt. Granaglione to the south of the springs (Ciancabilla et al. 2004, 2007). The Porretta Terme springs are long known for their peculiar healing properties, and the first local naturalistic reports documenting their properties (e.g., Capponi 1608) indeed claimed that medical exploitation started sometimes in the Etruscan period. Today, these waters are exploited by a Spa resort and have an important impact on the local economy. They are utilized for treatments concerning rehabilitation of the respiratory apparatus, peripheral vascular system, as well as deafness, gastrointestinal and dermatological diseases, and gynecological disorders (Facci et al. 1995).

In addition to the presence of spring waters, Porretta Terme is also known for another significant geological occurrence, i.e., the presence of shear and extension fissure veins hosting large euhedral quartz crystals (> 10 cm in size) with prismatic and/or skeletal habitus. The peculiarity of such occurrence is that a number of fissure veins outcrop exactly within the thermal spring area, and the large skeletal quartz crystals host fluid inclusions containing an aqueous fluid and a CH_4_-bearing fluid, i.e., similar to that of the water springs. The fissure quartz and its fluid inclusions were the focus of modern geological studies (Mullis 1988), although fissure veins and their large quartz crystals were investigated by a mineralogical point of view in the nineteenth century (Bombicci 1873; Gambari 1868). The large fluid inclusions described within the prismatic and skeletal quartz should be considered among the first documentations of fluid inclusions in the scientific literature (the term *quarzo aeroidro* was coined for the inclusion-bearing quartz).

In this study, we combine a geochemical dataset gathered for the spring waters, including the concentrations of major and minor water solutes, the chemical composition of dissolved gas species, and the isotopic composition of water (δ^18^O and δD), carbon (^13^C/^12^C ratios in TDIC, CO_2_ and CH_4_), with microthermometric properties of aqueous and CH_4_-bearing fluid inclusions from the Porretta quartz. The aim is to propose a comprehensive geochemical model on the genesis of the spring waters. This multidisciplinary geochemical approach is an attempt to constrain the origin of the thermal waters and the key secondary processes occurring in the spring area with the recent structural evolution of this region from the Apennines, which generated the quartz-bearing fissure veins. In this scenario, we consider the fissure veins and the H_2_O–CH_4_ fluid entrapped within the prismatic and hopper quartz as the product of hydrothermal fluid flow and mineral precipitation that was coeval with the spring activity in the geological past.

The geochemical data from cold springs emerging within and in the surroundings of the thermal area are also reported and discussed in order to evaluate the possible interaction between deep and shallow aquifers.

### Study area

**Fig. 2:**
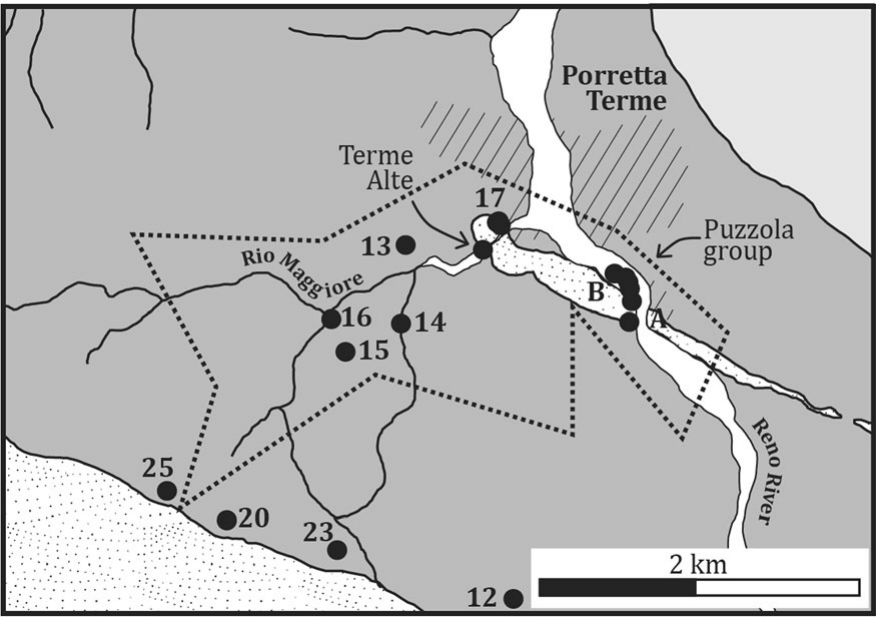
Geological sketch of the Porretta Terme area and local drainage basin. Symbols of tectonic–stratigraphic units as shown in Fig. 1. The dotted area marks the mining concession area of the Porretta Terme Spa resort. The numbered symbols mark the water–gas sampling locations of this study. The hatched area indicates the village of Porretta Terme. Letters A and B show the positions of the historical quarries where large hopper quartz crystals were found in the nineteenth century, i.e., Madonna del Ponte and Cava della Costa, respectively

The study area includes a large portion of the Alto Reno area (Figs. 1 and 2), which extends several kilometers from the area of the thermal springs. The exploitation history of the Porretta thermal baths dates back probably since the Roman times, as testified by a first-century AC marble sculpture found on the bed of the Rio Maggiore creek in 1888 and depicting the face of a lion, which marked the presence of an ancient bath (Facci et al. 1995). In the first half of the nineteenth century, the Lion-Ox resort (Terme Alte, Fig. 2) was built on the ancient thermal foundation and included several buildings, namely Leone, Bove, Marte, Sale, and Donzelle from the names of the associated springs. The recently built Spa resort, located along the Reno river and renovated in 2016, collects the thermal waters from the Terme Alte springs and those of the Puzzola group (Fig. 2), emerging SE of the Porretta village including the Puzzola, Maiocchi, Porretta Nuova, and Porretta Vecchia springs.

#### Geological setting and mineralogical features

Northern Apennines is a fold and thrust belt consisting of several tectonic–stratigraphic units emplaced toward N–NE from the Early Cretaceous to present in relation with the closure of the Piedmont-Ligurian Ocean and the collision of the European and Adria plates (Molli 2008). The lower structural level corresponds to the Tuscan (nappe) units consisting of a Paleozoic crystalline basement and Mesozoic carbonates (Triassic evaporite and dolostone). Foredeep basins developed in front of the northeastward migrating chain were filled by Late Oligocene–Miocene siliciclastic turbidite sequences (Ricci Lucchi 1986; Boccaletti et al. 1990) until sedimentation stopped due to the overthrusting Ligurian Units, consisting of highly tectonized ophiolites and a Jurassic to Eocene sedimentary cover, currently at the top of the Apennine nappe pile (Principi and Treves 1984).

The Sestola-Vidiciatico Tectonic Unit that hosts the study area (Figs. 1 and 2) developed during the early-middle Miocene as a regional-scale shear zone that was 200 km long and 200 m thick. It formed between the overthrusting Ligurian paleo-accretionary complex and the underthrusting Tuscan/Umbrian Units of the Adriatic continental margin (e.g., Vannucchi et al. 2008, and references therein). The Sestola-Vidiciatico Unit consists of distinct tectonically superposed subunits, which are hundreds of meters to kilometers wide and derived from the Ligurian accretionary complex and the slope deposits emplaced in its frontal part.

In this sector of the Apennines, CH_4_-rich gases discharged by mud volcanoes and gas seeps likely originate from both (i) the Marnoso Arenacea turbiditic formation overlying the Miocene basement and (ii) deeper Triassic rocks (Capozzi and Picotti 2002, 2010; Etiope et al. 2007).

In the Porretta area, a normal active fault system (Picotti and Pazzaglia 2008) separates the northern sector, characterized by the presence of the Ligurian nappe, and the southern sector, where Lower Miocene interbedded hard sandstones and shale mudstone crop out. The main reservoir of the local petroleum system is considered to be hosted within the Tertiary foredeep units (Macigno Cervarola), dated at 3 to 6 Ma (Ventura et al. 2001), which were affected by secondary porosity (Gargini et al. 2008).

The quartz–calcite fissure veins outcrop within a broad region that extends beyond Porretta Terme. Their relations with the regional and local lineaments are not completely defined, since the historical literature on the quartz–calcite fracture systems mainly focused on the mineralogical features (e.g., Bombicci 1873; Gambari 1868), while the geological documentation is rather poor. Historical data allowed identifying a > 50 km^2^ outcropping region of the quartz–calcite fissure veins, which extends, therefore, away from Porretta Terme. To the south of Porretta, fissure veins were reported at Pracchia, Monte Granaglione, and Lizzo (Fig. 1), while to the north of Porretta they were reported at Riola and Monteacuto Ragazza. This broad outcropping region suggests that the fissure veins are related to a significant regional event of deformation and fluid flow within this sector of the northern Apennine. Notably, hopper quartz fissures were historically documented within the thermal spring area of Porretta (Fig. 2).

**Fig. 3:**
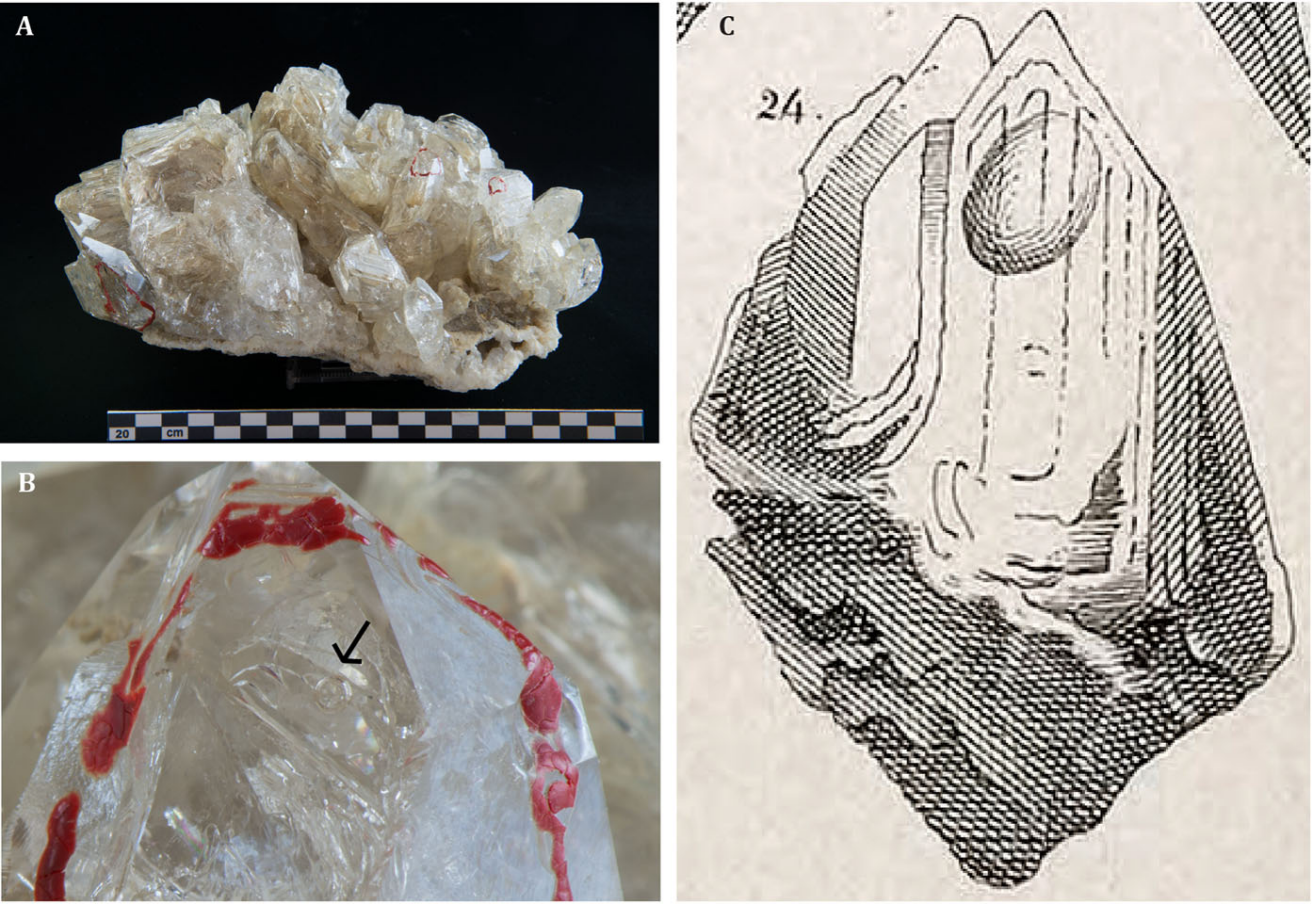
Hopper quartz from the shear and extension fissure veins of Porretta Terme. A. Large slab of hopper quartz from one of the first discovered fissure veins (Porretta Terme, Madonna del Ponte old quarry. Photograph: courtesy from Museo Bombicci, Università di Bologna, Italy). The red marks on the crystal locate the occurrence of large aqueous fluid inclusions. B. Detail of one marked crystal showing a large field inclusion (arrow points to vapor bubble. Photograph: courtesy from Museo Bombicci, Università di Bologna, Italy). C. Detail of one of the historical documentations of the hopper quartz from Porretta Terme (Gambari 1868) showing a crystal hosting a large fluid inclusion indicated by the round dark bubble

Quartz (Fig. 3) and calcite were reported as the most common fissure minerals, while clays (of undefined compositions), mixtures of oxides/hydroxides of ochraceus color, and minor Mg–Fe carbonates (mesitine) occur as minor phases within the fissures. These minerals were also documented as solid inclusions together with carbonaceous material within the quartz fluid inclusions (Bombicci 1873; Gambari 1868). Recent studies on the CH_4_-rich and H_2_O-rich fluid inclusion populations entrapped within prismatic and hopper fissure quartz by microthermometry, Raman spectroscopy, and gas chromatography constrained the conditions at which quartz crystals formed (Mullis 1988). The concomitant presence of CH_4_-rich and H_2_O-rich fluid inclusion types within the same primary growth bands of quartz, and in particular the abundance of the CH_4_-rich fluid within the hopper crystals, was interpreted as the products of a parent H_2_O–NaCl–CH_4_ fluid that was phase–separating at the time of quartz crystallization. Accordingly, the two distinct fluid types represent two end-member fluids entrapped within quartz while the parent fluid was effervescing, and the fissures were sealing. The CH_4_-rich fluid inclusion population (CH_4_: 87–96 mol%) contained minor proportions of CO_2_ and H_2_O and was interpreted as the vapor-rich end-member generated by effervescence. The H_2_O-rich population, which hosted a 0.5–0.6 wt% NaCl-equivalent aqueous solution and minor proportions of CH_4_ (1.5–1.7 mol%), represented the liquid-rich end-member. The estimated temperature of phase separation was determined in the 220–230 °C range and the pressure in the 200–210 MPa range, while fluid pressures as low as 55–190 MPa were calculated for some paragenetically late fluid inclusion populations entrapped within quartz. The important implication derived by the evidence for phase separation is the fact that, at least locally, the estimated fluid pressure must have approached lithostatic conditions at least locally (Becker et al. 2010; Roedder and Bodnar 1980). This represents an important constraint on the underground paleofluid regime of the Porretta drainage basin, the hopper quartz fissures representing the roots of a vertically extensive fracture set that experienced hydrothermal fluid flow in a regime of uplift and exhumation.

## Materials and methods

### Sampling and analysis of waters

Waters and dissolved gases (34 samples) were collected in March–June 2019 from 11 thermal springs (tw), 19 cold springs (cw), Rio Maggiore creek (r; 2 samples), and Reno river (r; 2 samples).

Outlet water temperature (T) and pH were measured in situ with a portable multiparametric instrument (Crison 2000). The chemical and isotopic analysis of waters and dissolved gases was carried out at the Laboratory of Fluid Geochemistry of the University of Florence (Italy), excepting those of (i) the ^18^O/^16^O and ^2^H/^1^H ratios of water (δ^18^O–H_2_O and δD–H_2_O, expressed as ‰vs. V-SMOW) that were performed at the Laboratory of Stable Isotopes of Parma (Italy), and (ii) the ^13^C/^12^C ratios in Total Dissolved Inorganic Carbon (δ^13^C-TDIC, expressed as ‰ vs. V-PDB) that were measured at the Laboratory of Stable Isotopes of CNR-IGG in Pisa (Italy). Waters were collected in high-density polyethylene (HDPE) bottles, as follows: 2 samples (50 mL) filtered (0.45 μm) and acidified with Suprapure HCl and HNO_3_ for the analysis of major cations and trace elements, respectively; 1 filtered sample (125 mL) for the analysis of anions; 1 unfiltered sample (50 mL), diluted (1:10) in situ, for the analysis of SiO_2_; 1 unfiltered sample (50 mL) for the analysis of δ^18^O–H_2_O and δD–H_2_O; 1 unfiltered sample (250 mL) for the analysis of δ^13^C–TDIC, after adding SrCl_2_ and NaOH to precipitate the dissolved carbonate species as SrCO_3_ (Caliro et al. 1999); 1 unfiltered sample (8 mL) in a plastic tube filled with 2 mL of a Cd–NH_4_ solution (Montegrossi et al. 2006) for the determination of the reduced sulfur species (H_2_S, HS^−^ and S^2−^, expressed as ΣS^2−^).

Total alkalinity (mostly consisting of HCO_3_^−^ with minor CO_3_^2−^) was determined by acidimetric titration (AT) using HCl 0.01 N and methyl orange as the indicator. Main anions (F^−^, Cl^−^, Br^−^, NO_3_^−^ and SO_4_^2−^) and cations (Ca^2+^, Mg^2+^, Na^+^, K^+^, and Li^+^) were analyzed by ion chromatography (IC) using Metrohm 761 and Metrohm 861 chromatographs, respectively. The contents of SO_4_^2−^ obtained by oxidation of ΣS^2−^ collected using the Cd-NH_4_ trap were determined by IC (Metrohm 761). Ammonia was measured by molecular spectrophotometry (MSP) using a Hach DR2100 instrument, whereas SiO_2_ was analyzed by spectrophotometry (SP) (Bencini and Martini 1979). The analytical errors for AT, MSP, SP, and IC were ≤ 5%. Trace elements (Mn, Fe, Ba, and As) were determined by Inductively Coupled Plasma Optical Emission Spectrometry (ICP-OES) using an Optima 8000 PerkinElmer spectrometer. The analytical error for ICP-OES was ≤ 10%.

The δ^18^O–H_2_O and δD–H_2_O values were determined by isotope ratio mass spectrometry (IRMS) using (i) a Gas Bench peripheral coupled with a Thermo Delta V mass spectrometer and (ii) a TC-EA peripheral interfaced with a Thermo Delta XP mass spectrometer, respectively. The analytical errors for IRMS were ± 0.1‰ and ± 1‰ for δ^18^O and δD, respectively. The δ^13^C-TDIC values were measured by Mass Spectrometry (MS) using a Finningan MAT252 instrument following the procedure described by Salata et al. (2000). Analytical uncertainty and reproducibility for MS were ± 0.05‰ and ± 0.1‰, respectively.

## Sampling and analysis of dissolved gases

Dissolved gases were collected as water samples in pre-evacuated and pre-weighed 250-mL glass flasks equipped with Thorion® valves, leaving a headspace of approximately 30% of the total flask volume (Capasso and Inguaggiato 1998). The analysis CO_2_, N_2_, (Ar + O_2_), and H_2_ in the headspace of the sampling flasks was carried out by gas chromatography (GC) using a Shimadzu 15A instrument equipped with a 5-m-long stainless steel column packed with Porapak 80/100 mesh and a Thermal Conductivity Detector (TCD). Argon and O_2_ were analyzed using a Thermo Focus gas chromatograph equipped with a 30-m-long capillary molecular sieve column and a TCD. Methane and C_2_–C_4_ hydrocarbons were determined by using a Shimadzu 14A gas chromatograph equipped with a Flame Ionization Detector (FID) and a 10-m-long stainless steel column packed with Chromosorb PAW 80/100 mesh coated with 23% SP 1700 (Vaselli et al. 2006). The analytical error for the GC analysis was ≤ 10%. The concentrations of the dissolved gases (in μmol/L) were given by the sum of n_i,g_, i.e., the moles of the *i* gas in the sampling flask headspace measured by GC, and n_i,l_, i.e., the moles of the *i* gas that remained in the water collected in the sampling flasks. The n_i,l_ values were calculated from the n_i,g_ ones by means of Henry’s law constants (Wilhelm et al. 1977), assuming that in the sampling flasks the separated gas phase was in equilibrium with the liquid.

The δ^13^C/^12^C ratios in CO_2_ and CH_4_ (δ^13^C–CO_2_ and δ^13^C–CH_4_, expressed as ‰ vs. V-PDB) were analyzed using a WSCRDS (Wavelength-Scanned Cavity Ring Down Spectroscopy) analyzer (Picarro G2201-i). In order to avoid interferences related to the presence of water vapor and H_2_S, the instrument inlet line was equipped with Drierite and copper traps. According to the operative ranges of the instrument, samples having CH_4_ concentrations > 0.5 mmol/mol were diluted using a N_2_–O_2_–Ar gas mixture. Analytical errors for WSCRDS were 0.16‰ and 1.15‰ for δ^13^C–CO_2_ and δ^13^C–CH_4_, respectively.

The measured δ^13^C–CO_2_ values of these gas aliquots (δ^13^C–CO_2meas_) were used to compute the δ^13^C values of dissolved CO_2_ according to the ε_1_ fractionation factor for the gas–water isotope equilibrium proposed by Zhang et al. (1995), as follows:

1 $$\varepsilon 1 = \delta 13{\text{C}} - {\text{CO}}_{2} - \delta 13{\text{C}} - {\text{CO}}_{{2{\text{meas}}}} = (0.0049 \times T) - 1.31$$

where the temperature (*T*) is in °C.

## Sampling and analysis of fluid inclusion samples

**Fig. 4:**
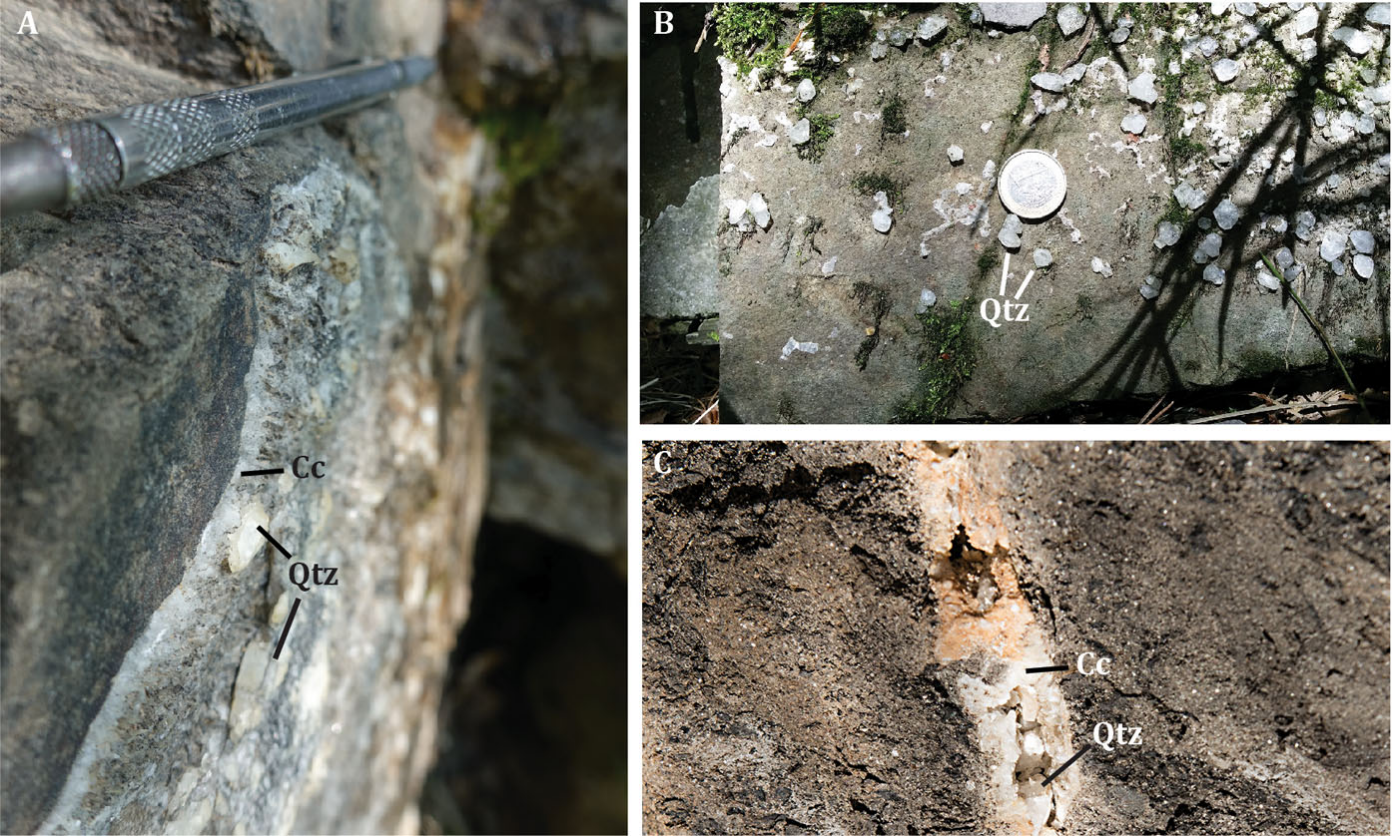
Field occurrence of the quartz–calcite fissure veins. ** a** Contact between the host sandstone (*Formazione di Stagno*) and a quartz (Qtz)—calcite (Cc) fissure at Case Calistri (cf. Fig. 1). To note the euhedral shape of both minerals within the fracture. ** b** Wall of a quartz fissure in which euhedral quartz grows attached to the walls (Case Calistri). ** c** Quartz–calcite fissure from the thermal spring area (Galleria delle Sorgenti, close to the Puzzola spring)

The quartz samples analyzed for this study were collected as part of an ongoing geological study focusing on the relations between the architecture of the recent brittle structures of the northern Apennines and the tectonic evolution of the orogenic prism. The euhedral quartz samples DDS 18-02 and DDS 18-08 were collected from two fracture sets (I and II) outcropping at Case Calistri (Fig. 1) and filled with the typical quartz–calcite assemblage of the Porretta Terme area (Fig. 4a, b). The fissures crosscut the greywackes of *Formazione di Stagno*. Quartz in these fissures is prismatic with no hopper texture. The fissures are both steeply dipping (> 70°) and strike both NE and SW, but with different azimuth angles (DDS 18-02: 40°. DDS 18-08: 20°). They belong to groups of quartz–calcite-filled fractures whose attitude and kinematic indicators can be interpreted as conjugated and recording an initial strike slip followed by a later extensional (mode I) component of motion (De Santis 2018). Identical quartz–calcite-filled fractures were also found within the thermal spring area (Fig. 4c).

The fluid inclusion study was exclusively conducted on “fluid inclusion assemblages” (i.e., FIA; Goldstein and Reynolds 1994), i.e., on petrographically discriminated, cogenetic groups of fluid inclusions located along trails or (less commonly) growth planes of the host mineral. By definition, FIAs are groups of inclusions trapped together at a specific stage of mineral formation and, as such, they give the highest level of confidence when characterizing the properties of trapped fluids and discriminating possible stages of post-entrapment re-equilibration (Bodnar 2003).

Seventy-three microthermometric measurements from 3 FIAs (FIA1 from DDS 18-08; FIAs 2–3 from DDS 18-02) hosted by euhedral quartz were carried out at the Department of Biological, Geological and Environmental Sciences of the University of Bologna using a Linkam THMSG 600 heating/freezing stage mounted on an Olympus BX51 petrographic microscope. The heating–freezing stage was calibrated using synthetic fluid inclusions at –56.6, 0.0, and 374 °C, corresponding to the melting of pure CO_2_, melting of pure H_2_O, and critical homogenization of pure H_2_O, respectively. Fluid inclusions of known composition containing a H_2_O–NaCl–CO_2_ fluid and having a melting T of the clathrate at ~7.5 °C were used to test the accuracy of measurements, which resulted being ~ 0.1 °C at these conditions. Reproducibility was determined with duplicate or triplicate measurements of individual inclusions and was better than 0.5 °C at low temperatures and 1 °C at high temperatures.

All low-temperature phase transitions were measured using the following standard procedure. Samples were first rapidly cooled to −180 °C in order to detect the potential formation of eutectic phases, salt hydrates, solid carbonic phase, clathrate, and ice. In all measurement sessions, the only phase transition we determined in all assemblages was the melting of the ice—*T*m(ice) and the total homogenization temperatures—*T*h(total). All phase transitions were measured using the cycling method described by Goldstein and Reynolds (1994), and care was taken in recording the minimum and maximum values for each assemblage.

In order to identify molecular species within the fluid inclusions of the quartz samples (DDS 18-02 and DDS 18-08), seven inclusions belonging to one-phase, vapor-rich FIAs were studied with a Raman spectrometer at the Department of Mathematical, Physical and Informatic Sciences of the University of Parma (Italy). This equipment consisted of a Jobin–Yvon Horiba LabRam spectrometer connected to a confocal microscope, a He–Ne laser (emission line: 632.8 nm), and a motorized stage. Spectral resolution of measurements was determined at 2 cm^−1^, and confocal aperture was regulated to obtain a spatial resolution (lateral and in depth) of 1–2 μm. Calibration was carried out using the 520.7 cm^−1^ peak of metallic Si, and spectra were acquired using a high magnification objective (100x, N.A. = 0.75). The 100–3600 cm^−1^ spectral range was scanned systematically to determine the presence of CO_2_, N_2_, CH_4_, and H_2_S, although the measurements were conducted at 1100–1800 cm^−1^ and 2500–3300 cm^−1^ since CO_2_ and CH_4_ were the only molecular species present in the fluid inclusions. Measurement times varied between 1 and 30 s and were combined with 3–30 accumulations. The power on the sample surface was nearly 1 mW, but the power on the analyzed inclusions had to be considered lower due to reflections and scattering. Analyses were carried out on the vapor bubbles of the fluid inclusions.

## Results

### Chemical and isotopic (δ^18^O–H_2_O, δD–H_2_O, and δ^13^C–TDIC) composition of waters

**Fig. 5:**
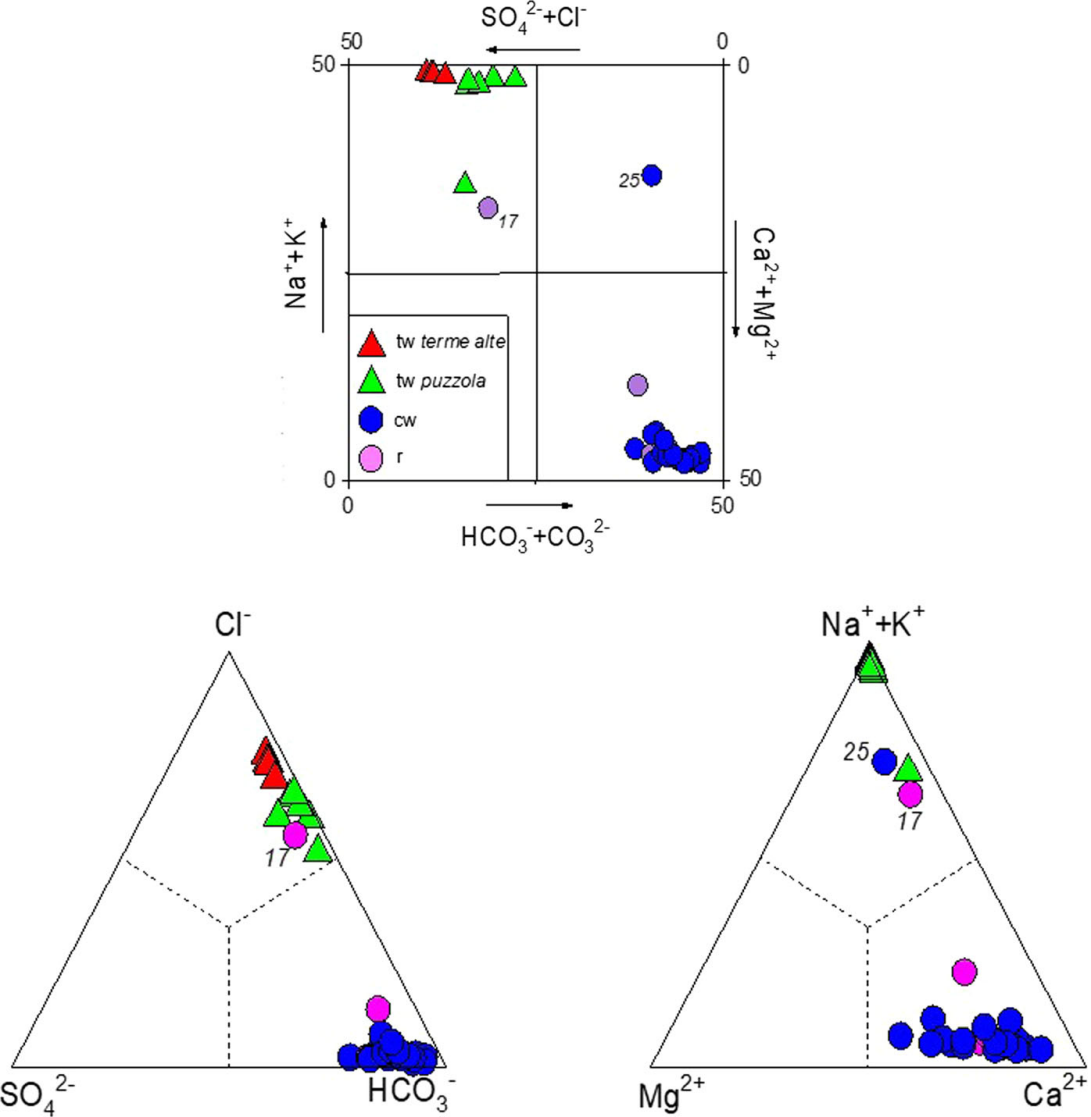
Square diagram (Langelier and Ludwig 1942), SO_4_^2−^–Cl^−^–HCO_3_^−^, and Ca^2+^–(Na^+^ + K^+^)–Mg^2+^ ternary diagrams for thermal (tw) and cold waters (cw) and rivers (r) from Porretta Terme. Red triangle, thermal waters from Terme Alte; green triangle, thermal waters from Puzzola; blue circle, cold waters; purple circle, rivers

Geographical coordinates, main chemical–physical parameters (outlet temperature, pH), and chemical composition (in mg/L) of the Porretta waters are reported in Table 1. Thermal waters (tw) from Terme Alte (Sale, Bove, Marte, Donzelle, and Donzelle Galleria) show the highest outlet temperatures (from 27.6 to 35.4 °C), pH values ranging from 6.98 to 7.82, while those of TDS are from 3,555 to 5,750 mg/L. The Puzzola springs (Porretta Vecchia, Maiocchi, Porretta Nuova, Laghetto Galleria, and Puzzola) display outlet temperatures from 19.1 to 28.5 °C, pH from 7.69 to 8.80, and pH and TDS values from 7.69 to 8.80 and from 1,180 to 2,344 mg/L, respectively. All thermal waters are characterized by a Na^+^–Cl^−^ composition (Fig. 5), with significant concentrations of HCO_3_^−^ (up to 1,190 mg/L) and, secondarily, SO_4_^2−^ (up to 201 mg/L). Cold waters (cw) and rivers (r) have temperatures < 18.1 °C, pH from 7.80 to 8.80, relatively low TDS values (from 254 to 789 mg/L), and a Ca^2+^–HCO_3_^−^ composition (Fig. 5). Two exceptions are represented by the Rio Maggiore Valle sample (*17*), showing a Na^+^–Cl^−^ composition as expected since it collects waters discharged from Terme Alte springs, and Mulino di Granaglione sample (*25*), which has a Na^+^–HCO_3_^−^ composition likely due to local anthropogenic contamination, as suggested by the relatively high pH values and Cl^−^ and NH_4_^+^ concentrations (Table 1).

**Table 1:** Geographic coordinates, altitude, outlet temperatures (in °C), pH, chemical composition (in mg/L), TDS values (in mg/L), δ^13^C-TDIC (as ‰ vs. V-PDB), δD-H_2_O and δ^18^O–H_2_O (‰ vs. V-SMOW) values of thermal and cold waters from flPorretta Terme

		Type	Date	E	N	Altitude	T	pH	HCO_3_^−^	CO_3_^2−^	F^−^
*1*	Sale	tw	07-03-2019	657,760	4,890,806	375	32.3	7.40	1160	1.5	6.9
*2*	Bove	tw	07-03-2019	657,734	4,890,820	375	29.0	7.41	1190	1.5	5.9
*3*	Marte	tw	07-03-2019	657,739	4,890,757	375	35.4	7.55	1020	1.8	6.0
*4*	Donzelle	tw	07-03-2019	657,651	4,890,789	375	30.3	7.45	1040	1.5	6.3
*5*	Donzelle Galleria	tw	07-03-2019	657,717	4,890,736	375	27.6	7.24	852	0.74	4.5
*6*	Porretta vecchia	tw	13-03-2019	657,755	4,890,784	360	28.5	7.82	790	2.6	4.2
*7*	Maiocchi	tw	13-03-2019	658,518	4,890,513	362	24.4	7.55	635	1.1	4.3
*8*	Porretta Nuova	tw	13-03-2019	658,508	4,890,566	362	26.6	7.70	782	2.0	5.0
*9*	Puzzola Nuova	tw	13-03-2019	658,566	4,890,552	361	25.0	7.59	616	1.2	3.9
*10*	Laghetto Galleria	tw	13-03-2019	658,530	4,890,544	360	19.1	7.91	570	2.3	4.5
*11*	Puzzola	tw	13-03-2019	658,588	4,890,516	360	19.2	6.98	321	0.15	1.1
*12*	Lustrola	cw	09-04-2019	657,934	4,888,604	610	14.5	8.05	510	2.9	0.23
*13*	Carriola	cw	09-04-2019	657,024	4,890,097	472	15.3	7.96	413	1.9	0.17
*14*	Torretta	cw	09-04-2019	657,456	4,890,235	445	13.8	7.80	422	1.3	0.15
*15*	Palareda	cw	09-04-2019	656,781	4,890,267	431	15.5	8.00	388	2.0	0.28
*16*	Rio Maggiore monte	r	09-04-2019	657,487	4,890,579	378	15.3	8.23	218	1.9	0.10
*17*	Rio Maggiore valle	r	09-04-2019	657,926	4,890,868	364	16.9	7.69	316	0.78	0.83
*18*	Reno monte	r	06-05-2019	657,021	4,884,900	491	18.1	8.45	168	2.4	0.10
*19*	Reno Silla	r	06-05-2019	658,204	4,892,898	326	19.9	8.38	212	2.6	0.11
*20*	Vettica	cw	06-05-2019	657,183	4,889,155	624	16.1	7.97	350	1.6	0.10
*21*	Fonte dell'Amore	cw	06-05-2019	663,060	4,886,946	515	13.9	8.10	315	2.0	0.09
*22*	Poggio di Badi	cw	06-05-2019	661,351	4,887,025	630	13.6	8.05	291	1.6	0.10
*23*	Varano	cw	06-05-2019	656,380	4,889,196	564	14.0	8.07	291	1.7	0.09
*24*	Docciola	cw	06-05-2019	653,873	4,895,695	710	11.6	8.52	240	4.0	0.09
*25*	Mulino di Granaglione	cw	13-06-2019	656,056	4,889,422	465	16.3	8.80	224	7.3	0.81
*26*	Fonte Perio	cw	13-06-2019	662,289	4,886,888	667	11.2	8.30	242	2.4	0.09
*27*	Fonte del 3	cw	13-06-2019	656,382	4,886,571	797	13.5	8.30	242	2.4	0.06
*28*	Fonte del 4	cw	13-06-2019	656,347	4,886,599	796	12.2	8.25	242	2.2	0.05
*29*	Fontana Foiado	cw	13-06-2019	656,314	4,886,145	765	16.9	8.21	215	1.8	0.06
*30*	Massovrana	cw	13-06-2019	662,539	4,886,975	602	12.4	8.30	211	2.1	0.07
*31*	Taviano	cw	13-06-2019	660,476	4,886,033	568	11.3	8.31	191	2.0	0.10
*32*	Pavana	cw	13-06-2019	660,153	4,886,302	494	12.1	8.20	182	1.5	0.14
*33*	Bellavalle	cw	13-06-2019	659,304	4,884,070	546	13.7	7.90	177	0.7	0.08
*34*	Limentra	cw	13-06-2019	657,714	4,880,772	653	13.5	8.39	169	2.1	0.07

		Type	Date	E	N	Altitude	Br^−^	NO_3_^−^	SO^2−^	Ca_2_^+^	Mg^2+^	Na^+^	K^+^	NH_4_^+^	SO_2_^−^	Ca^2+^	Mg^2+^
*1*	Sale	tw	07-03-2019	657,760	4,890,806	375	1.1	0.11	172	22	6.2	1840	71	9.5	172	22	6.2
*2*	Bove	tw	07-03-2019	657,734	4,890,820	375	1.3	0.40	172	21	7.9	1780	65	9.6	172	21	7.9
*3*	Marte	tw	07-03-2019	657,739	4,890,757	375	1.3	0.23	161	21	6.4	1650	56	8.1	161	21	6.4
*4*	Donzelle	tw	07-03-2019	657,651	4,890,789	375	1.2	0.31	165	22	6.9	1610	62	8.7	165	22	6.9
*5*	Donzelle Galleria	tw	07-03-2019	657,717	4,890,736	375	0.88	0.39	124	19	5.7	1190	39	5.6	124	19	5.7
*6*	Porretta vecchia	tw	13-03-2019	657,755	4,890,784	360	0.59	0.20	25	14	4.4	744	26	4.0	25	14	4.4
*7*	Maiocchi	tw	13-03-2019	658,518	4,890,513	362	0.50	0.20	58	23	4.9	647	27	3.4	58	23	4.9
*8*	Porretta Nuova	tw	13-03-2019	658,508	4,890,566	362	0.55	0.30	51	13	4.7	692	27	3.7	51	13	4.7
*9*	Puzzola Nuova	tw	13-03-2019	658,566	4,890,552	361	0.51	0.13	34	18	4.2	591	22	3.3	34	18	4.2
*10*	Laghetto Galleria	tw	13-03-2019	658,530	4,890,544	360	0.46	0.82	30	15	3.5	583	24	3.0	30	15	3.5
*11*	Puzzola	tw	13-03-2019	658,588	4,890,516	360	0.18	0.06	70	80	11	267	16	1.7	70	80	11
*12*	Lustrola	cw	09-04-2019	657,934	4,888,604	610	0.03	0.29	71	125	37	27	3.0	< 0.01	71	125	37
*13*	Carriola	cw	09-04-2019	657,024	4,890,097	472	0.02	0.36	89	88	38	13	2.8	< 0.01	89	88	38
*14*	Torretta	cw	09-04-2019	657,456	4,890,235	445	0.02	0.50	48	113	29	11	1.8	< 0.01	48	113	29
*15*	Palareda	cw	09-04-2019	656,781	4,890,267	431	0.03	0.01	48	92	25	10	3.0	0.01	48	92	25
*16*	Rio Maggiore monte	r	09-04-2019	657,487	4,890,579	378	0.01	0.89	36	60	11	5.4	1.1	0.01	36	60	11
*17*	Rio Maggiore valle	r	09-04-2019	657,926	4,890,868	364	0.14	2.6	46	71	12	194	8.9	0.44	46	71	12
*18*	Reno monte	r	06-05-2019	657,021	4,884,900	491	0.01	1.3	17	49	7.5	5.5	1.0	0.01	17	49	7.5
*19*	Reno Silla	r	06-05-2019	658,204	4,892,898	326	0.07	1.1	19	52	8.5	22	1.8	< 0.01	19	52	8.5
*20*	Vettica	cw	06-05-2019	657,183	4,889,155	624	< 0.01	0.19	22	77	25	8.0	1.0	< 0.01	22	77	25
*21*	Fonte dell'Amore	cw	06-05-2019	663,060	4,886,946	515	0.02	4.0	15	97	7.9	6.1	0.72	< 0.01	15	97	7.9
*22*	Poggio di Badi	cw	06-05-2019	661,351	4,887,025	630	0.01	0.10	9	84	4.7	4.6	0.55	< 0.01	9	84	4.7
*23*	Varano	cw	06-05-2019	656,380	4,889,196	564	< 0.01	0.02	11	77	7.2	7.0	0.66	< 0.01	11	77	7.2
*24*	Docciola	cw	06-05-2019	653,873	4,895,695	710	0.03	1.3	38	75	7.0	10	3.6	< 0.01	38	75	7.0
*25*	Mulino di Granaglione	cw	13-06-2019	656,056	4,889,422	465	0.12	0.01	24	16	5.4	79	2.1	0.49	24	16	5.4
*26*	Fonte Perio	cw	13-06-2019	662,289	4,886,888	667	0.05	0.65	20	63	14	5.0	1.0	< 0.01	20	63	14
*27*	Fonte del 3	cw	13-06-2019	656,382	4,886,571	797	0.01	1.0	19	68	7.2	4.5	0.65	0.01	19	68	7.2
*28*	Fonte del 4	cw	13-06-2019	656,347	4,886,599	796	< 0.01	1.2	14	65	8.4	4.6	0.71	< 0.01	14	65	8.4
*29*	Fontana Foiado	cw	13-06-2019	656,314	4,886,145	765	< 0.01	2.4	35	63	9.2	3.7	1.1	0.01	35	63	9.2
*30*	Massovrana	cw	13-06-2019	662,539	4,886,975	602	0.05	0.68	14	62	6.3	3.5	0.57	< 0.01	14	62	6.3
*31*	Taviano	cw	13-06-2019	660,476	4,886,033	568	0.06	1.6	23	49	11	4.9	0.91	0.01	23	49	11
*32*	Pavana	cw	13-06-2019	660,153	4,886,302	494	0.03	1.3	18	52	6.2	4.0	0.78	< 0.01	18	52	6.2
*33*	Bellavalle	cw	13-06-2019	659,304	4,884,070	546	0.01	1.6	15	50	7.1	4.2	0.84	0.01	15	50	7.1
*34*	Limentra	cw	13-06-2019	657,714	4,880,772	653	0.02	1.7	15	44	6.9	6.3	1.1	< 0.01	15	44	6.9

		Type	Date	E	N	Altitude	Li^+^	SIO_2_	δS^2−^	Ba	Fe	Mn	As	TDS	δ^18^O–H_2_O	δD-H_2_O	δ^13^C-TDIC
*1*	Sale	tw	07-03-2019	657,760	4,890,806	375	3.3	68	10	37	0.29	0.07	0.083	5753	−7.06	−46.3	−13.1
*2*	Bove	tw	07-03-2019	657,734	4,890,820	375	3.2	73	10	31	0.39	0.08	0.078	5508	−7.06	−46.3	−12.7
*3*	Marte	tw	07-03-2019	657,739	4,890,757	375	2.7	65	12	27	0.22	0.07	0.050	4917	−7.24	−46.9	−8.47
*4*	Donzelle	tw	07-03-2019	657,651	4,890,789	375	2.9	71	11	33	0.33	0.08	0.079	4878	−7.52	−47.5	−19.4
*5*	Donzelle Galleria	tw	07-03-2019	657,717	4,890,736	375	1.7	62	10	21	0.24	0.07	0.049	3584	−7.80	−49.9	−16.5
*6*	Porretta vecchia	tw	13-03-2019	657,755	4,890,784	360	1.3	65	14	18	0.08	0.05	0.045	2344	−7.81	−49.4	−17.5
*7*	Maiocchi	tw	13−03-2019	658,518	4,890,513	362	1.0	50	24	15	0.11	0.16	0.046	2187	−7.75	−49.9	−12.0
*8*	Porretta Nuova	tw	13-03-2019	658,508	4,890,566	362	1.2	61	15	17	0.25	0.06	0.062	2125	−7.86	−49.6	−11.3
*9*	Puzzola Nuova	tw	13-03-2019	658,566	4,890,552	361	1.1	53	19	16	0.15	0.14	0.069	1962	−7.86	−49.6	−14.6
*10*	Laghetto Galleria	tw	13-03-2019	658,530	4,890,544	360	0.98	60	14	15	0.06	0.08	0.093	1934	−7.88	−49.7	−11.2
*11*	Puzzola	tw	13-03-2019	658,588	4,890,516	360	0.43	40	51	6.5	1.3	0.56	0.11	1180	−7.45	−47.6	−17.4
*12*	Lustrola	cw	09-04-2019	657,934	4,888,604	610	0.045	22	24	0.06	0.005	0.005	< 0.001	813	−8.11	−50.9	−17.4
*13*	Carriola	cw	09-04-2019	657,024	4,890,097	472	0.036	33	25	0.07	0.015	0.002	0.015	679	−7.76	−48.5	−15.4
*14*	Torretta	cw	09-04-2019	657,456	4,890,235	445	0.024	29	16	0.12	0.004	0.005	< 0.001	649	−7.77	−48.2	−11.7
*15*	Palareda	cw	09-04-2019	656,781	4,890,267	431	0.019	35	17	0.11	0.003	0.006	< 0.001	596	−7.79	−48.1	−15.5
*16*	Rio Maggiore monte	r	09-04-2019	657,487	4,890,579	378	0.012	22	11	0.05	0.002	0.007	< 0.001	348	−8.91	−56.9	−16.1
*17*	Rio Maggiore valle	r	09-04-2019	657,926	4,890,868	364	0.034	45	12	0.42	0.002	0.008	< 0.001	941	−8.55	−54.9	−15.4
*18*	Reno monte	r	06-05-2019	657,021	4,884,900	491	0.008	27	< 1	0.06	0.014	0.001	< 0.001	257	−8.55	−55.1	−16.3
*19*	Reno Silla	r	06-05-2019	658,204	4,892,898	326	0.035	28	< 1	0.09	0.012	0.003	< 0.001	341	−8.05	−50.2	−15.7
*20*	Vettica	cw	06-05-2019	657,183	4,889,155	624	0.017	23	< 1	0.06	0.003	0.005	< 0.001	487	−8.12	−51.5	−14.6
*21*	Fonte dell'Amore	cw	06-05-2019	663,060	4,886,946	515	0.007	36	< 1	0.10	0.012	< 0.001	< 0.001	455	−8.09	−50.3	−16.9
*22*	Poggio di Badi	cw	06-05-2019	661,351	4,887,025	630	0.008	17	< 1	0.13	0.015	< 0.001	< 0.001	400	−8.08	−49.7	−17.2
*23*	Varano	cw	06-05-2019	656,380	4,889,196	564	0.010	32	< 1	0.08	0.005	0.005	< 0.001	398	−7.93	−49.1	−8.82
*24*	Docciola	cw	06-05-2019	653,873	4,895,695	710	0.023	31	< 1	0.03	0.004	0.005	< 0.001	384	−8.28	−52.4	−17.7
*25*	Mulino di Granaglione	cw	13-06-2019	656,056	4,889,422	465	0.099	21	< 1	0.28	0.009	0.009	< 0.001	372	−7.75	−48.5	−17.1
*26*	Fonte Perio	cw	13-06-2019	662,289	4,886,888	667	0.012	28	< 1	0.08	0.011	< 0.001	< 0.001	352	−8.27	−53.2	−13.2
*27*	Fonte del 3	cw	13-06-2019	656,382	4,886,571	797	0.009	30	< 1	0.11	0.012	< 0.001	< 0.001	349	−8.29	−53.6	−13.5
*28*	Fonte del 4	cw	13-06-2019	656,347	4,886,599	796	0.008	24	< 1	0.10	0.015	< 0.001	< 0.001	341	−8.34	−52.9	−13.2
*29*	Fontana Foiado	cw	13-06-2019	656,314	4,886,145	765	0.009	30	< 1	0.06	0.013	< 0.001	< 0.001	334	−8.33	−52.8	−12.9
*30*	Massovrana	cw	13-06-2019	662,539	4,886,975	602	0.008	33	< 1	0.06	0.001	0.005	< 0.001	305	−8.18	−51.9	−13.5
*31*	Taviano	cw	13-06-2019	660,476	4,886,033	568	0.010	28	< 1	0.04	0.016	< 0.001	< 0.001	289	−8.02	−50.5	−12.3
*32*	Pavana	cw	13-06-2019	660,153	4,886,302	494	0.007	26	< 1	0.05	0.003	0.005	< 0.001	271	−7.86	−48.2	−12.9
*33*	Bellavalle	cw	13-06-2019	659,304	4,884,070	546	0.008	37	< 1	0.05	0.015	< 0.001	< 0.001	262	−7.87	−48.1	−11.9
*34*	Limentra	cw	13-06-2019	657,714	4,880,772	653	0.008	18	< 1	0.07	0.014	0.001	< 0.001	254	−8.11	−51.5	−12.8

As far as the minor compounds are concerned, Br^−^ concentrations follow those of Cl^−^, as the concentrations of these anions in the thermal waters are 1–2 orders of magnitude higher than those of the cold ones. Similarly, the SiO_2_, NH_4_^+^, F^−^ and Li^+^ concentrations from the thermal waters (up to 73, 9.6, 6.9 and 3.3 mg/L) are significantly higher than those from the cold waters (up to 45, 0.49, 0.83 and 0.045 mg/L). The ΣS^2−^ values in thermal waters from Terme Alte range from 10 to 13 mg/L, whilst those of the Puzzola waters and cold waters from the thermal Spa resort (including Rio Maggiore creek; Fig. 2) are slightly higher (from 14 to 51 and from 11 to 25 mg/L).

The concentrations of trace elements (in mg/L) and the δ^18^O–H_2_O, δD–H_2_O and δ^13^C-TDIC values are reported in Table 1. The concentrations of all the selected trace elements in thermal waters are relatively high (up to 37, 1.3, 0.56, and 0.11 mg/L for Ba, Fe, Mn, and As, respectively), whereas the corresponding concentrations measured in the cold waters are 1–2 orders of magnitude lower or below the detection limits (As in most cases). The δ^18^O–H_2_O and δD-H_2_O values of the thermal waters range from −7.88‰ to −7.06‰ and from −49.7‰ to −46.3‰ vs. V−SMOW, respectively, whereas those of the cold waters range from −8.91‰ to −7.75‰ and from −56.9‰ to −48.1‰ vs. V-SMOW, respectively. The δ^13^C-TDIC values range from −8.47‰ to −17.5‰ vs. V-PDB, without any clear difference between thermal and cold waters.

The Saturation Indexes (SI) were computed with the PHREEQC v2.18 code (Parkhurst and Appelo 1999) using the llnl.dat database at the measured outlet temperatures and solute concentrations reported in Table 1. As reported in Table 2, all the thermal waters are oversaturated with respect to quartz, chalcedony, and calcite (except *5* and *11*) and undersaturated with respect to anhydrite and gypsum. Most thermal waters (*1*, *2*, *3*, *4*, *6*, and *8*) are oversaturated with respect to dolomite and undersaturated with respect to aragonite (*1*, *2*, *4*, *5*, *7*, *8*, *9*, *10*, and *11* samples).

**Table 2:** Saturation Indexes (SIs) with respect to anhydrite, aragonite, calcite, dolomite, gypsum, quartz, and chalcedony calculated for thermal waters from Porretta Terme at the measured outlet temperatures and solutes concentrations reported in Table 1 using the PHREEQC v2.18 code (Parkhurst and Appelo 1999) and the llnl.dat database

		Anhydrite	Aragonite	Calcite	Dolomite	Gypsum	Quartz	Chalcedony
*1*	Sale	−2.61	−0.06	0.08	0.04	−2.39	0.61	0.20
*2*	Bove	−2.64	−0.09	0.05	0.07	−2.39	0.69	0.27
*3*	Marte	−2.57	0.09	0.22	0.38	−2.38	0.54	0.14
*4*	Donzelle	−2.59	−0.05	0.09	0.09	−2.35	0.65	0.24
*5*	Donzelle Galleria	−2.71	−0.39	−0.25	−0.64	−2.43	0.63	0.21
*6*	Porretta vecchia	−3.41	0.09	0.23	0.36	−3.14	0.63	0.21
*7*	Maiocchi	−2.85	−0.11	0.03	−0.26	−2.54	0.58	0.15
*8*	Porretta Nuova	−3.12	−0.07	0.07	0.07	−2.83	0.64	0.21
*9*	Puzzola Nuova	−3.13	−0.14	0.00	−0.29	−2.83	0.60	0.17
*10*	Laghetto Galleria	−3.33	−0.03	0.12	−0.12	−2.96	0.74	0.29
*11*	Puzzola	−2.18	−0.48	−0.33	−1.26	−1.81	0.56	0.12

### Chemical and isotopic (δ^13^C–CO_2_ and δ^13^C–CH_4_) composition of dissolved gases

The chemical composition of dissolved gases (in μmol/L) and the δ^13^C–CO_2_ and δ^13^C–CH_4_ values are reported in Table 3. Gas samples from thermal springs are CH_4_–dominated (from 856 to 1053 μmol/L) with significant concentrations of CO_2_ (up to 184 μmol/L) and N_2_ (up to 159 μmol/L). Argon and O_2_ concentrations are up to 4.0 and 0.64 μmol/L, respectively, whereas C_2_–C_4_ alkanes mostly consist of C_2_H_6_ (up to 7.5 μmol/L) followed by C_3_H_8_ (up to 0.66 μmol/L), i-C_4_H_10_ (up to 0.33 μmol/L) and n-C_4_H_10_ (up to 0.42 μμmol/L). Hydrogen shows relatively low concentrations, from 0.011 to 0.025 μmol/L. Dissolved gases in cold waters and rivers are largely dominated by N_2_ (from 601 to 800 μmol/L) and O_2_ (from 90 to 243 μmol/L), with minor concentrations of Ar (up to 20 μmol/L) and CO_2_ (up to 8.5 μmol/L). Methane was detected in few samples mostly located in the proximity of the thermal Spa (*12*, *13*, *16*, *17*, *19*, *22* and *25*) at concentrations ranging from 2.5 to 68 μmol/L. The δ^13^C–CO_2_ values from the thermal waters show a relatively wide variability (from −21.0‰ to −8.7‰ vs. V-PDB), whereas those of cold waters range from −17.0‰ to −22.1‰ vs. V-PDB), except that of sample 17 (Rio Maggiore Valle; −13.4‰ vs. V-PDB). The δ^13^C–CH_4_ values of the thermal springs are significantly higher (from −22‰ to −30‰ vs. V-PDB) with respect of those of the cold springs (from −56‰ to −50‰ vs. V-PDB). The anomalously high δ^13^C–CH_4_ value of sample 5 (Donzelle Galleria; 3.5‰ vs. V-PDB) is likely due to secondary CH_4_ consumption.

**Table 3:** Chemical composition (in mmol/mol) and δ^13^C–CH_4_ and δ^13^C–CO_2_ values of gases dissolved in thermal and cold waters from Porretta Terme

		CH_4_	CO_2_	N_2_	Ar	O_2_	H_2_	C_2_H_6_	C_3_H_8_	i-C_4_H_10_	n-C_4_H_10_	δ^13^C-CH_4_	δ^13^C-CO_2_
*1*	Sale	899	147	159	4.0	0.35	< 0.01	4.6	0.26	0.15	0.16	−28	−8.7
*2*	Bove	967	184	30	0.82	0.64	0.019	1.3	0.15	0.07	0.09	−28	−8.7
*3*	Marte	992	123	26	0.67	0.13	0.017	6.8	0.36	0.21	0.25	−28	−9.1
*4*	Donzelle	1053	71	75	1.8	0.25	0.011	5.3	0.46	0.23	0.31	−22	−14.7
*5*	Donzelle Galleria	1,016	71	64	1.6	0.38	0.022	5.3	0.38	0.16	0.19	3.5	−19.0
*6*	Porretta vecchia	1016	76	53	1.1	0.058	0.013	6.7	0.51	0.24	0.27	−30	−10.5
*7*	Maiocchi	1,008	97	31	0.75	0.18	0.018	7.0	0.55	0.30	0.42	−30	−13.0
*8*	Porretta Nuova	1,052	103	33	0.81	0.16	0.014	5.4	0.25	0.13	0.16	−31	−11.0
*9*	Puzzola Nuova	1,009	90	57	1.1	0.33	0.014	5.9	0.43	0.22	0.28	−30	−12.8
*10*	Laghetto Galleria	856	144	77	1.9	0.64	< 0.01	1.4	0.16	0.12	0.13	−30	−8.0
*11*	Puzzola	996	108	72	1.8	0.059	0.025	7.5	0.66	0.33	0.42	−29	−21.0
*12*	Lustrola	19	6.0	637	15	211	< 0.01	0.1	< 0.1	< 0.1	< 0.1	−50	−20.4
*13*	Carriola	2.7	2.2	682	17	243	< 0.01	< 0.1	< 0.1	< 0.1	< 0.1	−50	−18.3
*14*	Torretta	< 1	1.8	700	18	158	< 0.01	< 0.1	< 0.1	< 0.1	< 0.1	n.d	−20.5
*15*	Palareda	< 1	1.3	661	16	134	< 0.01	< 0.1	< 0.1	< 0.1	< 0.1	n.d	−20.4
*16*	Rio Maggiore monte	3.9	1.6	709	17	183	< 0.01	< 0.1	< 0.1	< 0.1	< 0.1	−50	−17.0
*17*	Rio Maggiore valle	38	7.4	735	16	169	< 0.01	0.15	< 0.1	< 0.1	< 0.1	−52	−13.4
*18*	Reno monte	< 1	1.6	623	16	224	< 0.01	< 0.1	< 0.1	< 0.1	< 0.1	n.d	−18.0
*19*	Reno Silla	21	8.5	669	17	208	< 0.01	0.11	< 0.1	< 0.1	< 0.1	−56	−19.0
*20*	Vettica	< 1	1.6	690	17	146	< 0.01	< 0.1	< 0.1	< 0.1	< 0.1	n.d	−19.7
*21*	Fonte dell'Amore	< 1	3.1	663	15	132	< 0.01	< 0.1	< 0.1	< 0.1	< 0.1	n.d	−21.6
*22*	Poggio di Badi	2.5	2.0	711	17	234	< 0.01	< 0.1	< 0.1	< 0.1	< 0.1	−52	−21.8
*23*	Varano	< 1	1.5	621	15	200	< 0.01	< 0.1	< 0.1	< 0.1	< 0.1	n.d	−22.1
*24*	Docciola	< 1	1.8	593	15	213	< 0.01	< 0.1	< 0.1	< 0.1	< 0.1	n.d	−20.5
*25*	Mulino di Granaglione	68	11	800	20	90	< 0.01	0.31	< 0.1	< 0.1	< 0.1	−52	−12.5
*26*	Fonte Perio	< 1	2.0	702	17	94	< 0.01	< 0.1	< 0.1	< 0.1	< 0.1	n.d	−19.3
*27*	Fonte del 3	< 1	1.5	601	14	217	< 0.01	< 0.1	< 0.1	< 0.1	< 0.1	n.d	−19.0
*28*	Fonte del 4	< 1	1.6	667	16	176	< 0.01	< 0.1	< 0.1	< 0.1	< 0.1	n.d	−19.4
*29*	Fontana Foiado	< 1	1.4	602	14	224	< 0.01	< 0.1	< 0.1	< 0.1	< 0.1	n.d	−20.0
*30*	Massovrana	< 1	2.6	601	15	222	< 0.01	< 0.1	< 0.1	< 0.1	< 0.1	n.d	−19.0
*31*	Taviano	< 1	1.2	605	15	205	< 0.01	< 0.1	< 0.1	< 0.1	< 0.1	n.d	−18.0
*32*	Pavana	< 1	2.2	606	15	203	< 0.01	< 0.1	< 0.1	< 0.1	< 0.1	n.d	−17.0
*33*	Bellavalle	< 1	1.8	604	15	215	< 0.01	< 0.1	< 0.1	< 0.1	< 0.1	n.d	−19.0
*34*	Limentra	< 1	1.4	615	15	207	< 0.01	< 0.1	< 0.1	< 0.1	< 0.1	n.d	−18.0

### Fluid inclusions

**Fig. 6:**
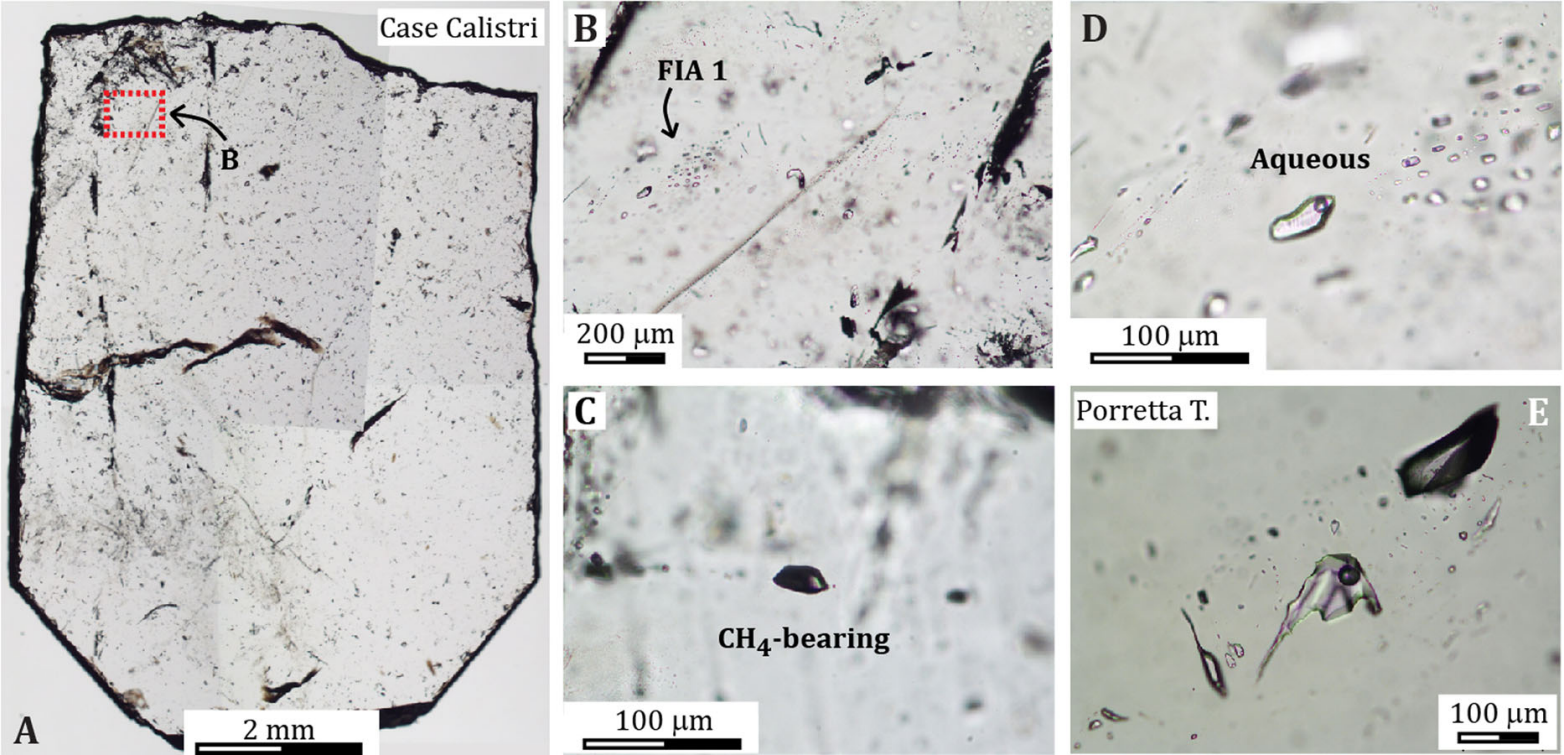
Characteristic textures and fluid inclusion assemblages from the Porretta Terme area. **a** Photocomposition of prismatic quartz from the Casa Calistri quartz–calcite fissure (sample DDS 18-08). The red square marks the position of panel B. **b** Detail of the pseudo-secondary trail of aqueous FIA 1 from sample DDS 18-08. **c** Methane-bearing (vapor-rich) trail of dark fluid inclusions from sample DDS 18-02. **d** Detail of panel B showing the two-phase (L–V) inclusions of FIA 1 and their constant L/V ratio. **e** Simultaneous occurrence of large (> 100 μm in size) aqueous and CH_4_-bearing fluid inclusions within a trail from a large hopper quartz from Porretta Terme (effervescing fluid)

At laboratory temperature, the Case Calistri fissure quartz (Figs. 4a and 6a) hosts two types of FIAs, namely an aqueous fluid within trails or clusters of two-phase (liquid–vapor; hereafter L and V, respectively) inclusions having constant L/V ratio (Fig. 6b, d), and a unary, CH_4_-bearing fluid made of trails or clusters of dark, vapor-rich inclusions (Fig. 6c). These inclusions are similar to those found within the hopper crystals of Porretta Terme, in which large aqueous and CH_4_-bearing inclusions were systematically recognized. Nevertheless, it is worth mentioning that the large hopper crystals from Porretta host both aqueous and CH_4_-bearing inclusions within the same trails or assemblages (Fig. 6e). Such textural difference is important because it has implications on the homogeneous vs. heterogeneous state of the Porretta and Case Calistri fluids at the time of their entrapment (see below).

**Fig. 7:**
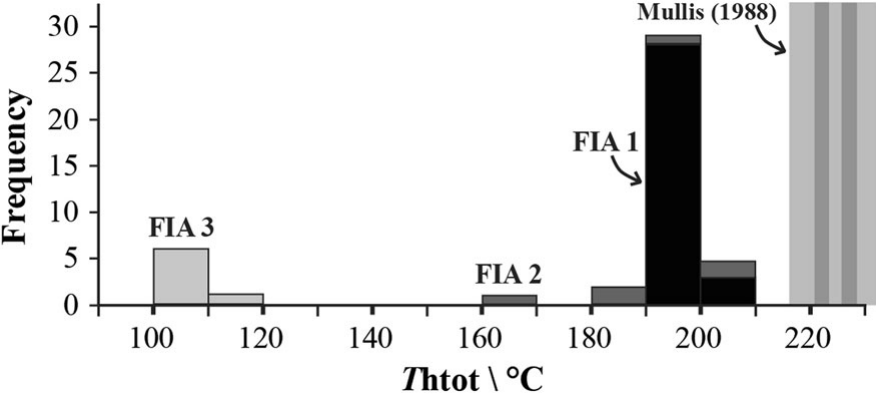
Total homogenization temperatures of the studied aqueous fluid inclusion assemblages from the fissure quartz of Case Calistri. The temperature intervals of total homogenization are very narrow for the 3 assemblages (FIA 1: 199 ± 1 °C; FIA 2: 192 ± 15 °C; FIA 3: 105 ± 5 °C). The *T* intervals reported to the right are mean values and ranges of *T*htot measured by Mullis (1988) for the phase-separating fluid from the hopper quartz of Porretta Terme

The three studied FIAs host aqueous inclusions with a constant phase ratio (L/V) and consistent microthermometric properties. The *T*m(ice) of FIA 1 is equal to −0.9 ± 0.1 °C, which corresponds to a calculated 1.5 ± 0.1 wt% NaCl-equivalent salinity. Ice melting was not visible in all the studied inclusions; consequently, no bulk salinity data were calculated for the entire dataset. The melting of a solid at -77.5 °C documented in FIA 2 was interpreted as the melting T of a carbonic phase. The *T*h(tot) of the three FIAs systematically occurs by bubble disappearance and ranges from 105 to 200 °C. However, the *T*htot values of individual assemblages are very narrow and equal to 199 ± 1 °C, 192 ± 15, and 105 ± 5 (Fig. 7), showing that the three assemblages of entrapped fluids were in a homogeneous state at the time of entrapment.

Five CH_4_-bearing fluid inclusions entrapped within FIAs of the two studied samples (e.g., Fig. 6c) were analyzed by Raman Spectrometry in order to identify molecular components in addition to CH_4_ in the assemblages. Results show that all these inclusions consist exclusively of CH_4_, which shows a characteristic peak at 2934 cm^−1^ wavenumber. In one analysis, a peak at about 1,609 cm^−1^ was recorded and interpreted as the detection of amorphous elemental carbon.

## Discussion

### Primary sources and chemical–physical processes controlling the chemistry of waters

**Fig. 8:**
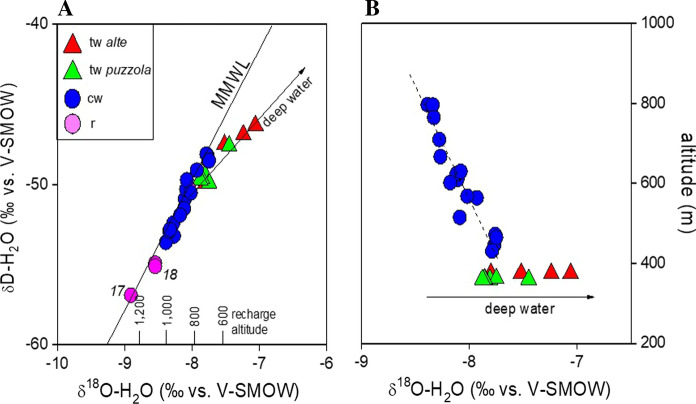
**a** δD-H_2_O versus δ^18^O–H_2_O (as ‰ vs. V-SMOW) and **b** altitude (m) versus δ^18^O–H_2_O binary diagrams for thermal and cold waters from Porretta Terme. The Mediterranean Meteoric Water Line (MMWL; Gat and Carmi 1970) is also reported. Symbols as shown in Fig. 5

**Fig. 9:**
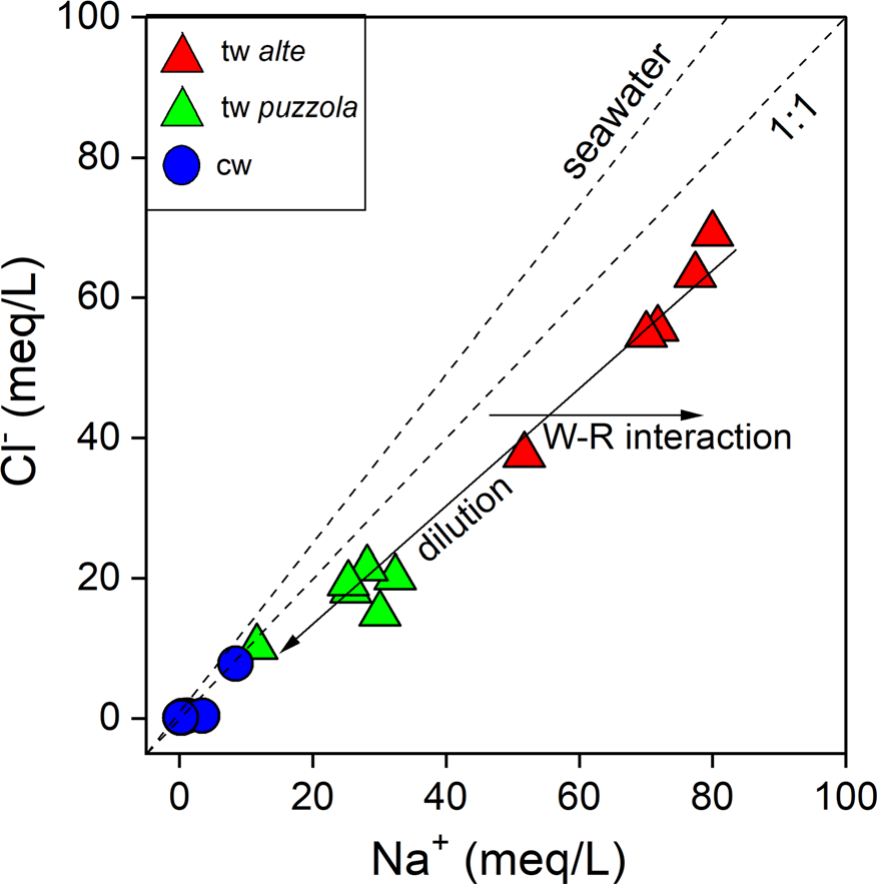
Cl^−^ versus Na^+^ (both in meq/L) binary diagram for thermal and cold waters from Porretta Terme. The two dotted lines are stoichiometric and seawater Cl^−^/Na^+^ ratios, respectively. Symbols as shown in Fig. 5

As shown in the δD versus δ^18^O diagram (Fig. 8a), the cold waters plot along the Mediterranean Meteoric Water Line (MMWL; Gat and Carmi 1970). This suggests that the meteoric recharge permeated at 700 to 1000 m a.s.l., consistently with the altitudes of the mountains surrounding the study area. The δD versus δ^18^O data also indicate recharge zones at higher altitudes for the Reno river (*18*) and for Rio Maggiore creek (*17*). This is consistent with the known source areas of those rivers, which are located close to the S. Marcello Piteglio village in Tuscany (at 1024 m a.s.l.) and at ~6 km W of Porretta (> 1200 m a.s.l), respectively. The δ^18^O–H_2_O versus altitude diagram (Fig. 8b) confirms the strong relation between altitude and the isotopic signature of the cold waters, whose alignment depicts a mean ^18^O–^16^O fractionation gradient of about 0.2‰/100 m, in agreement with that proposed for central Italy by Longinelli and Selmo (2003) and Minissale and Vaselli (2011). The thermal waters are characterized by significant enrichments of both D-H_2_O and ^18^O–H_2_O with respect to MMWL (Fig. 8a), which cannot be ascribed to vapor loss that would produce a similar trend, since all the thermal springs are captured at depth to favor the water supply to the Spa resort. It is worth noting that the intercept between the MMWL and the thermal water alignment corresponds to a recharge altitude > 800 m a.s.l. (Fig. 8a). This implies that the most probable recharge area for the Porretta thermal system is to be located to the SW of Porretta village, in agreement with the hypothesis proposed by Ciancabilla et al. (2004, 2007). The D- and ^18^O-enrichment trend (Fig. 8a) may be caused by the addition of isotopically heavy deep waters. This process was described by previous workers (e.g., Capozzi and Picotti 2010), who considered the Porretta waters as partly derived by seawater entrapped in a reservoir within the Cervarola sandstones at > 1,000 m depth (Fig. 1). This interpretation is consistent with the relatively high Na^+^ and Cl^−^ concentrations, although the Na^+^/Cl^−^ mol-ratios are higher than that of seawater (Fig. 9), likely due to a prolonged interaction with the sedimentary rocks. The relatively high concentrations of minor and trace elements measured in the thermal waters (e.g., Li^+^, Fe, Mn, Ba, and As; Tables 1 and 2), a geochemical feature commonly shown by hydrothermal fluids (Stauffer and Thompson 1984; Kaasalainen and Stefánsson 2012; Göb et al. 2013), support this hypothesis. This supports the occurrence of a deep hydrothermal reservoir feeding the Porretta thermal water discharges. In particular, the concentrations of As in the thermal waters largely exceed the limit concentration for human consumption (0.01 mg/L WHO 2011;). It is worth noting that the Cl^−^/Br^−^ ratios typically characterizing seawater-originated oilfield brines (100–300; Vengosh and Pankratov 1998) are significantly lower than those of the Porretta thermal waters (from 960 to 2,240), which weakens a marine origin of the deep waters. In fact, salt dissolution of from evaporitic formations would better explain these high Cl^−^/Br^−^ ratios (Freeman 2007; Alcala and Custodio 2008). This is consistent with the genetic model of Ciancabilla and Bonoli (2010), who suggested that the highly saline end-member fluid originates at great depth (down to 3,000 m) within the Triassic evaporitic formations. A similar fluid source was also invoked for mud volcanoes in this sector of the Apennines (Pieri 2001; Bonini et al. 2013). Hence, we propose that the Porretta Terme waters result from the deep infiltration of local meteoric waters down to a depth at which the interaction with the Triassic evaporitic formations takes place, producing a highly saline hydrothermal aquifer.

**Fig. 10:**
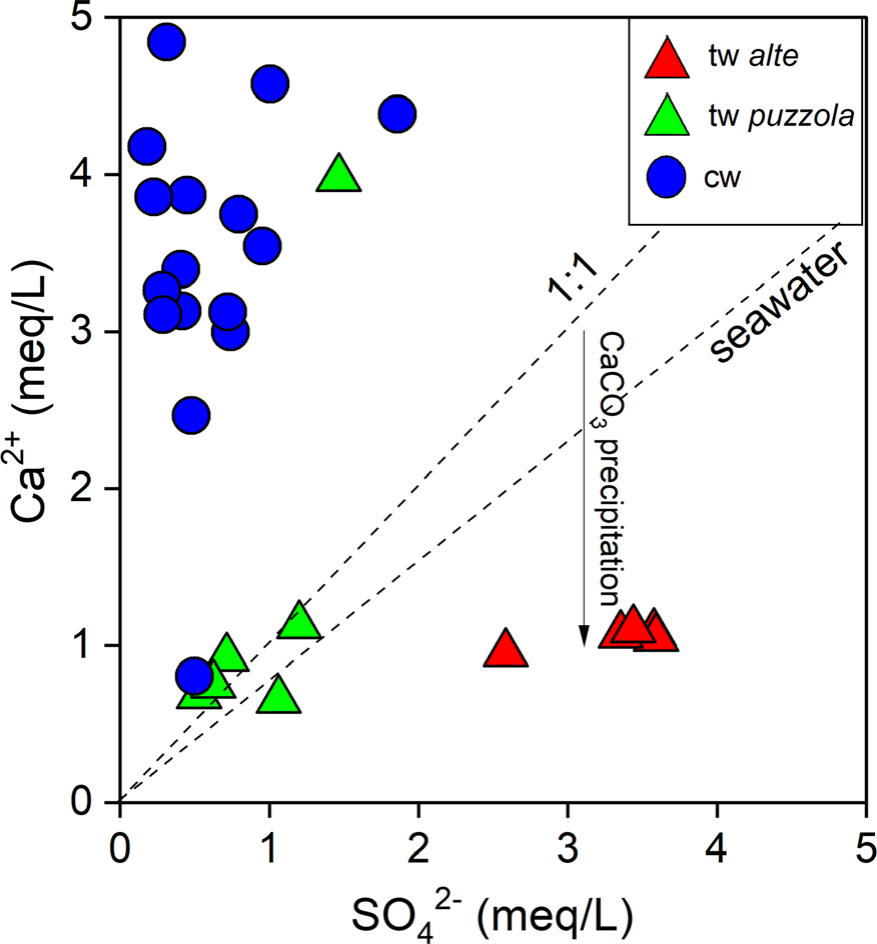
Ca^2+^ versus SO_4_^2−^ (both in meq/L) binary diagram for thermal and cold waters from Porretta Terme. The two dotted lines refer to the stoichiometric and seawater Cl^−^/Na^+^ ratios, respectively. Symbols as shown in Fig. 5

During their uprising, favored by fractures related to the extensional tectonic regime currently developing in the area (Minissale 2000; Bonini 2013), the hydrothermal fluids mix with deep waters within the organic-rich Upper Oligocene–Lower Miocene turbidites (Porretta-Suviana and Cervarola formations), further modifying its chemical and isotopic composition. At this stage, the presence of trapped seawater cannot be excluded. The low concentrations of Ca^2+^ in the thermal springs, i.e., lower than those expected for fluids deriving from dissolution of evaporite salts and seawater (Fig. 10), are likely due to calcite precipitation, as supported by the positive SI values shown in Table 2. Similarly, the precipitation of dolomite (Table 2) controls the Mg^2+^ concentrations in the thermal waters, which are comparable or even lower than those of the cold waters (Table 1), although Mg^2+^ may be also incorporated into clay and sheet silicate lattices (Giggenbach 1988; Gunnlaugsson and Einarsson 1989). The precipitation of carbonates mostly depends on the relatively high HCO_3_^−^ concentrations (Table 1), which are likely caused by HCO_3_^−^ production from degradation of organic matter (Drever 1997), a process that also produces NH_4_^+^ (Table 1) and sulfate reduction (Berner et al. 1970). The low δ^13^C-TDIC values measured in the thermal waters (Table 1) are consistent with this hypothesis. On the other hand, water–rock interactions typically lead to the observed enrichments in Na^+^ (Fig. 9) and K^+^ (Table 1). Hence, the chemical features of the deep-originated waters are strongly modified by different secondary chemical–physical processes, mostly occurring at reducing conditions in the Miocene sedimentary formations.

**Fig. 11:**
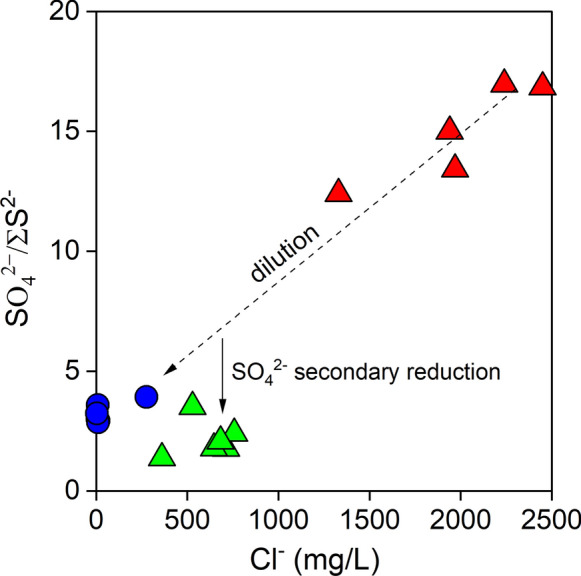
SO4^2−^/∑S^2−^ versus Cl^−^ (in mg/L) binary diagram for thermal and cold waters from Porretta Terme. Symbols as shown in Fig. 5

We stress that the most peculiar feature of the Porretta Spa resort is the availability of two distinct types of waters, characterized by different SO_4_^2−^/∑S^2−^ ratios (Fig. 11). The Puzzola group owes its name to the rotten egg smell emitted by the discharged waters, typically indicating the presence of reduced sulfur gases (H_2_S). The relatively high concentrations of ∑S^2−^ shown by these springs were interpreted by Ciancabilla and Bonoli (2010) as related to SO_4_^2−^-reducing microbial activity in the presence of CH_4_ as electron donor (Cassanini 2019 and references therein), according to the following reaction:

2 $${\text{CH}}_{4} + {\text{SO}}_{4}^{2} \to {\text{HCO}}_{3} + {\text{HS}} + {\text{H}}_{2} {\text{O}}$$

Sulfur-reducing bacteria are added to the Puzzola uprising deep waters at relatively shallow depths, where they mix with the waters from the Reno river, as suggested by the observed relationship between the TDS values of the Puzzola springs and the river flow (Ciancabilla and Bonoli 2010). This also explains the relatively low TDS values of the Puzzola waters with respect to those of Terme Alte (Fig. 5; Table 1).

A unique signature is provided to the Porretta thermal waters by the stable carbon isotope composition of CH_4_, i.e., the main dissolved gas compound produced at great depth by organic matter degradation. The δ^13^C–CH_4_ values (Table 3) and the CH_4_/(C_2_H_6_ + C_3_H_8_) ratios (from 122 to 664) in the thermal waters are indeed undistinguishable from those shown by thermogenic gases (Schoell 1980, 1988; Whiticar 1999; Etiope et al. 2009). Conversely, the measured δ^13^C–CO_2_ values (except that of sample *5* that is likely affected by shallow gas inputs) are significantly less negative than those typical of organic CO_2_, e.g., deriving from kerogen decarboxylation (from −15 to −20‰ V-PDB; Sano and Marty 1995), suggesting a contribution of isotopically heavier CO_2_ produced by thermometamorphism of limestone (δ^13^C–CO_2_ from −2 to + 2‰ V-PDB; Craig 1963). This further supports the occurrence of fluid contributions from the deep Triassic evaporites, although they are partially masked by chemical–physical processes and fluid addition occurring in the Miocene formations. To check the hypothesis of a double deep fluid source (i.e., from the Miocene and Triassic formations), the measured δ^13^C-TDIC values of the thermal waters, which are mostly depending on the origin of HCO_3_^−^ (i.e., the main C-bearing ionic species), are compared to those (δ^13^C-TDIC_calc_) computed on the basis of the measured δ^13^C–CO_2_ values (Table 4), according to the empirical equation suggested by Zhang et al. (1995), as follows:

3 $$\delta {^{13}}{\text{C - TDIC}}_{{{\text{calc}}}} = \delta {^{13}}{\text{C}} - {\text{CO}}_{2(g)} + {\text{H}}_{2} {\text{CO}}_{3} /{\text{TDIC}} \times \varepsilon_{1} + {\text{HCO}}_{3} /{\text{TDIC}} \times \varepsilon_{2} + {\text{CO}}_{3}^{2 - } {\text{/TDIC}} \times \varepsilon_{{3}}$$

We solved Eq. (3) considering the equilibrium molar ratios of aqueous carbon species at sampling temperature and pH, computed with the PHREEQC code, and the isotope fractionation factors ε_1_ (between H_2_CO_3_ and aqueous CO_2_), ε_2_ (between HCO_3_^−^ and aqueous CO_2_), and ε_2_ (between CO_3_^2−^ and aqueous CO_2_) (Deuser and Degens 1967; Mook et al. 1974). As shown in Table 1, all the δ^13^C-TDIC_calc_ values are strongly less negative than the measured ones, indicating that the isotopic signature of TDIC and CO_2_ values are not interdependent from each other, as expected if these compounds would have a common source. Hence, the processes occurring at reducing conditions (degradation of organic matter and sulfate reduction) within the organic-rich Miocene formations are responsible for the production of most HCO_3_^−^, whereas CO_2_ partially originates at greater depth from reactions involving the Triassic carbonates.

The low TDS values (Table 1) and the Ca^2+^-HCO_3_^−^ composition (Fig. 5) of the cold springs represent a clear indication of a relatively short hydrological pattern. This is supported by several evidences, such as (i) the lack of significant D- and ^18^O-enrichments (Fig. 8a), (ii) the relatively low concentrations of minor (e.g., NH_4_^+^ and Li^+^; Table 1) and trace elements (Table 1), and (iii) the air-dominated composition of the dissolved gases (Table 3). Further hints are also provided by both the δ^13^C–CH_4_ values (Table 3), corresponding to those typical of gases from microbial activity, and the δ^13^C–CO_2_ values (Table 3), in the range of CO_2_ from plant–root respiration and aerobic decay of organic matter (Cerling et al. 1991). The lack of clues of deep fluid contribution in the cold springs, excepting for those located near the thermal area (*12*, *13*, *14*, and *15*; Fig. 1), demonstrates that the uprising hydrothermal fluids are tectonically controlled up to the surface. This might represent the main geological factor that is responsible for the confinement of this water flow system, which preserved its quality over a geological time.

### The Porretta Terme paleofluid

The fluid inclusion assemblages entrapped within the euhedral quartz from Case Calistri constrain the physical–chemical properties of the paleofluid that generated the fracture sets. The presence within the quartz of two-phase, aqueous FIAs with constant phase ratios, and homogenizing consistently by bubble disappearance together with unary, CH_4_–bearing FIAs shows that two types of fluids were present within the fracture sets at the time of their filling. One of these was aqueous in composition, while the other was CH_4_–rich. The aqueous fluid was homogeneous at the time of its entrapment within the vein quartz, it had *T*h(tot) within the 100–200 °C range (minimum entrapment T; Fig. 7) and a NaCl-equivalent salinity of about 1.5 wt%. These properties correspond to those of a hydrothermal fluid that was precipitating quartz while flowing and cooling within the fracturing systems (Fournier 1985). Compared with the typical salinity of endogenous hydrothermal fluids forming mineral deposits (Heinrich and Candela 2014) or to that of oil field waters (Wilkinson et al. 2009), the bulk salinity of the aqueous fluid is low.

The common occurrence of CH_4_-bearing FIAs adjacent to aqueous FIAs within the same crystal (Fig. 6c) suggests that the methane-rich fluid derived from either unmixing from a deep, parent H_2_O–NaCl–CH_4_ fluid during maturation and cracking of organic matter or unmixing of the parent fluid due to isothermal decompression as a result of fracture opening (Mullis 1988). Both hypotheses are consistent with the microthermometric data. Notably, the highest *T*h(tot) ranges determined for the Case Calistri samples (Fig. 7) are 20–30 °C lower than the trapping temperatures (220–230 °C) estimated for the phase-separating (effervescing) hydrothermal fluid entrapped within the large hopper quartz of Porretta Terme. Such similarity suggests that the high-T fluid of Case Calistri was probably close to phase separation at the onset of fracture growth and that hopper crystals could not develop because effervescence, i.e., the conditions for which hopper growth is possible from an emulsion of liquid and vapor phases (Mullis 1988), did not occur in that location.

Entrapment temperatures of the Case Calistri fluid can be estimated by combining the calculated isochore of the aqueous fluid (not shown) with pressure corrections that take into account the maximum sedimentary and/or tectonic burial of the fractures at the time of fluid flow, e.g., using a combination of apatite fission track data, clay mineral assemblages, and vitrinite reflectance data. Considering a burial of ~3 km (corresponding to a lithostatic pressure of ~74 MPa) estimated by Caricchi et al. (2015) S of Porretta Terme as representative of our study area, an entrapment T in the 235–245 °C range is calculated for the Case Calistri fluid. If a burial of 5.5 km (corresponding to a lithostatic pressure of 135 MPa), estimated by Zattin (1998) for the Marnoso-Arenacea Formation of Porretta Terme, is taken into account, the calculated entrapment T is 270–280 °C. We stress that the estimated lithostatic load determined with these independent data is 3–5.5 km, therefore significantly lower than the 8–9 km of burial calculated by Mullis (1988) using the *T*h(tot) of the phase-separating fluid inclusions. The reason for such discrepancy can be found in the use of the H_2_O–CO_2_–CH_4_ equation of state used by Mullis (1988), which does not consider the presence of NaCl and therefore it ignores the salting-out effects occurring in H_2_O–NaCl–CH_4_ fluids at high temperatures (Cramer 1982).

### Past and present fluid reservoirs at Porretta Terme

The physical–chemical properties of the Porretta-Case Calistri paleofluid gathered from fluid inclusion data cannot directly be compared with those characterizing the present-day thermal fluid of Porretta. Notwithstanding, the spatial association of the deep-originated H_2_O–NaCl and CH_4_-rich hydrothermal fluids found in quartz–calcite fissure veins with those of the present-day reservoir feeding the Porretta Terme thermal springs cannot be considered coincidental but genetically linked. We propose that the structural setting that led to the production of the quartz–calcite fissures had a role in determining the recent permeability structure of the Porretta area, which ultimately controlled the local aquifer recharge and the locations of the thermal springs. If correct, this genetic link implies that the first thermal occurrences at Porretta was related to the development of the quartz fissures, the latter being dependent on the timing of the NW–SE extension indicated by the Case Calistri fractures. Thus, the fluid entrapped in the fissure quartz might represent the vestiges of the deep and hot, CH_4_-rich, Na^+^–Cl^−^ fluids produced by the interaction of meteoric waters with the Triassic and Miocene formations.

In principle, temperature estimations of the Porretta thermal waters based on the classic equilibrium reactions among ionic solutes are a challenging exercise due to the multiple fluid sources and the secondary processes envisaged in the area. As a first attempt, temperatures up to 92 °C are obtained based on chalcedony solubility (Arnorsson et al. 1983). Considering the present-day geothermal gradient of 30 °C/km, this temperature indicates that the equilibrium was attained at a depth of ~2.5 km, which approaches the 3–5.5-km depth interval estimated for the paleofluid entrapment. However, the temperatures provided by this geothermometer are likely underestimated, since the Porretta thermal waters are affected by dilution processes (Fig. 9). The Mg^2+^–Li^+^ pair, which is commonly considered a suitable geothermometer for waters circulating in sedimentary basins hosting oil production systems (Kharaka and Mariner 1989), cannot be used here because the concentrations of Mg^2+^ in the Porretta thermal waters are likely depending on secondary processes, such as dolomite deposition (Table 2) and reactions with silicates. Instead, the temperatures estimated with the empirical Na^+^–Li^+^ geothermometer (Kharaka et al. 1982) are 165–179 °C. Although these equilibrium temperatures should be taken with caution because small differences in Li^+^ concentrations in the samples would generate large variations in the calculated temperatures, we highlight that they would imply a fluid reservoir at a depth exceeding 5 km, which is consistent with the 3–5.5 km depth interval estimated for the Case Calistri paleofluid entrapment. Moreover, the Na–K geothermometer based on the equilibrium between Na- and K-feldspars (Giggenbach 1988) provides a similar temperature range (160–170 °C).

## Conclusions

At Porretta Terme, the use of a comprehensive set of chemical and isotopic data from present-day fluids in conjunction with physical–chemical properties of paleofluids from fluid inclusion data from well-constrained fracture sets proves being effective in constraining the primary fluid source regions and shallow secondary processes. The meteoric recharge area is located to the SW of Porretta village, as indicated by the δD and δ^18^O values, whereas the relatively high Cl^−^/Br^−^ ratios and δ^13^C–CO_2_ values suggest that the main hydrothermal reservoir is hosted within Triassic evaporite formations. The thermal fluids, uprising under the strict control of vertically extensive fault systems, interact with Miocene formations, where thermogenic CH_4_ production occurs. Deep-originated waters emerging close to Reno river are affected by mixing with the shallow aquifer rich in sulfate-reducing microbial populations. As a result of such hydrological cycle, the Spa resort may exploit two distinct types of waters, i.e., those rich in bromide and iodide and those rich in sulfide. Such a unique set of chemical compositions within the thermal area gives the opportunity to carry out a large variety of healing therapies. Out of the mining concession of the Porretta Terme resort, the shallow aquifer is characterized by lack of thermogenic CH_4_ and thermometamorphic CO_2_, low TDS values and low concentrations of NH_4_^+^, Li and trace elements, suggesting that no deep fluid inputs occur. Thus, those cold springs may represent an important drinking water resource for the area.

Our proposed genetic relations between the hydrothermal paleofluid of the quartz–calcite fissures the present-day thermal springs imply that the onset of thermal spring activity in the area is probably comparable to that of the NW–SE-directed crustal extension that accommodated the Case Calistri fracture set. If such hypothesis is true, the recharge/discharge cycle is long lasting and will remain so until crustal extension will modify the cycle significantly.

## References

[CR1] Alcala FJ, Custodio E (2008). Using the Cl/Br ratio as a tracer to identify the origin of salinity in aquifers in Spain and Portugal. Journal of Hydrology.

[CR2] Arnorsson S, Gunnlaugsson E, Svavarsson H (1983). The chemistry of geothermal waters in Iceland. III. Chemical geothermometry in geothermal investigations. Geochimica et Cosmochimica Acta.

[CR3] Becker, S. P., Eichhubl, P., Laubach, S. E., Reed, R. M., Lander, R. H., & Bodnar, R. J. (2010). A 48 m.y. history of fracture opening, temperature, and fluid pressure: Cretaceous Travis Peak Formation, East Texas basin. *Geological Society of America Bulletin, 122*(7–8), 1081–1093.

[CR4] Bencini A, Martini M (1979). Variazioni del contenuto di silice nelle acque freatiche di vulcano. Rendiconti della Società Italiana di Mineralogia e Petrologia.

[CR5] Berner RA, Scott MR, Thomlinson C (1970). Carbonate alkalinity in the pore waters of anoxic marine sediments. Limnology and Oceanography.

[CR6] Bertolini G, Gorgoni C (2001). La lavina di Roncovetro (Vedriano, Comune di Canossa, Provincia di Reggio Emilia). Quaderni di Geologia Applicata.

[CR7] Boccaletti M, Elter P, Guazzone G (1971). Plate tectonic models for the development of the Western Alps and Northern Apennines. Nature Physical Sciences.

[CR8] Boccaletti M, Ciaranfi N, Cosentino D, Deiana G, Gelati R, Lentini F, Massari F, Moratti G, Pescatore T, Ricci Lucchi F, Tortorici L (1990). Palinspastic restoration and paleogeographic reconstruction of the peri-Tyrrhenian area during the Neogene. Palaeogeography, Palaeoclimatology, Palaeoecology.

[CR9] Bodnar, R. J. (2003). *Re-equilibration of fluid inclusions (pp. 213–230).* In: Samson, I., Anderson, A., & Marshall, D. (Eds.), Fluid inclusions: Analysis and Interpretation. Mineralogical Association of Canada, Vancouver, Canada,

[CR10] Bombicci L (1873). Descrizione della mineralogia generale della Provincia di Bologna. Memorie dell'Accademia delle Scienze dell'Istitituto di Bologna.

[CR11] Bonoli, A., & Ciancabilla, F. (1995). Le acque minerali di Porretta terme. *Quarry and Construction*, 61–66. (in Italian).

[CR12] Bonini M (2013). Fluid seepage variability across the external Northern Apennines (Italy): structural controls with seismotectonic and geodynamic implications. Tectonophysics.

[CR13] Boschetti T, Toscani L, Shouakar-Stash O, Iacumin P, Venturelli G, Mucchino C, Frape SK (2011). Salt waters of the Northern Apennine Foredeep Basin (Italy): origin and evolution. Aquatic Geochemistry.

[CR14] Caliro S, Panichi C, Stanzione D (1999). Variation in the total dissolved carbon isotope composition of thermal waters of the Island of Ischia (Italy/) and its implications for volcanic surveillance. Journal of Volcanology and Geothermal Research.

[CR15] Capasso G, Inguaggiato S (1998). A simple method for the determination of dissolved gases in natural waters. Applied Geochemistry.

[CR16] Capozzi R, Picotti V (2002). Fluid migration and origin of a mud volcano in the northern Apennines (Italy): the role of deeply rooted normal faults. Terra Nova.

[CR17] Capozzi, R., & Picotti, V. (2010). Spontaneous fluid emissions in the Northern Apennines: geochemistry, structures and implications for the petroleum system. In Goffey, G. P., Craig, J., Needham, T. & Scott, R. (Eds.), *Hydrocarbons in Contractional Belts (pp.115–135).* Geological Society, London, Special Publications, 348.

[CR18] Capponi, P. (1608). Libro della Medicina delle acque Porrettane diviso in cinque trattati. Manuscript no. 1260, Biblioteca Comunale dell’Archiginnasio, Bologna. (in Italian).

[CR19] Caricchi C, Aldega L, Corrado S (2015). Reconstruction of maximum burial along the Northern Apennines thrust wedge (Italy) by indicators of thermal exposure and modeling. GSA Bulletin.

[CR20] Cassanini C (2019). Anaerobic oxidation of methane coupled to the reduction of different sulfur compounds as electron acceptors in bioreactors.

[CR21] Cerling TE, Solomon DK, Quade J, Bowman JR (1991). On the isotopic composition of carbon in soil carbon dioxide. Geochimica et Cosmochimica Acta.

[CR22] Cervi F, Borgatti L, Martinelli G, Ronchetti F (2014). Evidence of deep-water inflow in a tectonic window of the northern Apennines. Environmental Earth Sciences.

[CR23] Ciancabilla F, Bonoli A (2010). Nuovi approfondimenti sulla termalizzazione e mineralizzazione delle acque termominerali di Porretta Terme. *Il Geologo*. Journal of Ordine dei Geologi dell’Emilia Romagna.

[CR24] Ciancabilla N, Borgia GC, Bruni R, Ciancabilla F, Palmieri S, Vicari L (2004). Le sorgenti dell’Alta valle del Reno (Appennino Bolognese): nuovi elementi per approfondire la genesi dei movimenti gravitativi profondi nei terreni argillitici caoticizzati dell’appennino Tosco-Emiliano. *Il Geologo*. Journal of Ordine dei Geologi dell’Emilia Romagna.

[CR25] Ciancabilla N, Ditta M, Italiano F, Martinelli G (2007). The Porretta thermal springs (Northern Apennines): seismogenic structures and long-term geochemical monitoring. Annales of Geophysics.

[CR26] Craig, H. (1963). *The isotopic geochemistry of water and carbon in geothermal areas.*. In: Tongiorgi, E. (Ed.), Nuclear geology on geothermal areas. CNR (Italian Council for Research) (pp. 17–54), Spoleto, Italy.

[CR27] Cramer SD (1982). The solubility of methane, carbon dioxide, and oxygen in brines from 0° to 300 °C.

[CR28] De Santis, D. (2018). *Analisi strutturale e di inclusioni fluide di un sistema di fratture a quarzo-calcite dell’area di Porretta Terme (Bologna)*. (pp. 1–26). Bachelor Thesis, University of Bologna, Bachelor Thesis (in Italian).

[CR29] Deuser WG, Degens ET (1967). Carbon isotope fractionation in the system CO2 (gas)-CO_2_ (aqueous)-HCO_3_ (aqueous). Nature.

[CR30] Drever JI (1997). The Geochemistry of Natural Waters.

[CR31] Duchi V, Venturelli G, Boccasavia I, Bonicolini F, Ferrari C, Poli D (2005). Studio geochimico dei fluidi dell’Appennino Tosco-Emiliano-Romagnolo. Bollettino della Società Geologica Italiana.

[CR32] Etiope, G., Martinelli, G., Caracausi, A., & Italiano, F. (2007). Methane seeps and mud volcanoes in Italy: gas origin, fractionation and emission to the atmosphere. *Geophysical Research Letters, 34*. 10.1029/2007GL030341

[CR34] Etiope G, Feyzullayev A, Baciu CL (2009). Terrestrial methane seeps and mud volcanoes: a global perspective of gas origin. Marine and Petroleum Geology.

[CR35] Faccenna, C., Piromallo, C., Crespo-Blanc, A., Jolivet, L., & Rossetti, F. (2004). Lateral slab deformation and the origin of the western Mediterranean arcs. *Tectonics, 23*(1), TC1012.

[CR36] Facci, M., Guidanti, A., & Zagnoni, R. (1995). *Le Terme di Porretta nella Storia e nella Medicina.* Nuèter Editions.

[CR37] Fournier, R. O. (1985). *The behavior of silica in hydrothermal solutions. (pp. 45–72)*. In: Gerger, B. R., & Bethke P. M. (Eds.), Geology and Geochemistry of Epithermal Systems, Reviews in Economic Geology, 2.

[CR38] Freeman JT (2007). The use of bromide and chloride mass rations to differentiate salt-dissolution and formation brines in shallow groundwater of the Western Canadian Sedimentary Basin. Hydrogeology Journal.

[CR39] Gambari L (1868). Descrizione dei quarzi di Porretta. Annuario della Società dei Naturalisti di Modena.

[CR40] Gargini A, Vincenzi V, Piccinini L, Zuppi GM, Canuti P (2008). Groundwater flow systems in turbidites of the northern Apennines (Italy): natural discharge and high speed railway tunnel drainage. Hydrogeology Journal.

[CR41] Gat JR, Carmi I (1970). Evolution of the isotopic composition of atmospheric waters in the Mediterranean Sea area. Journal of Geophysical Research.

[CR42] Giggenbach WF (1988). Geothermal solute equilibria. Derivation of Na–K–Mg–Ca geoindicators. Geochimica et Cosmochimica Acta.

[CR43] Göb S, Loges A, Nolde N, Bau M, Jacob DE, Markl G (2013). Major and trace element compositions (including REE) of mineral, thermal, mine and surface waters in SW Germany and implications for water–rock interaction. Applied Geochemistry.

[CR44] Goldstein, R. H., & Reynolds, T. J. (1994). *Fluid inclusion microthermometry.* In: Goldstein, R. H. (Ed.), Systematics of fluid inclusions in diagenetic minerals, SEPM Short Course 31. SEPM (Society for Sedimentary Geology) (pp. 87–121), Tulsa, Oklahoma.

[CR45] Gunnlaugsson E, Einarsson A (1989). Magnesium-silicate scaling in mixture of geothermal water and deareated fresh water in a district heating system. Geothermics.

[CR46] Heinrich, C. A., Candela, P. A. (2014). *Fluids and ore formation in the Earth's Crust.* In: Holland, H. D., & Turekian, K. K. (Eds.), Treatise on Geochemistry (Second Edition) (pp. 1–28). Elsevier, Oxford.

[CR47] Kaasalainen H, Stefansson A (2012). The chemistry of trace elements in surface geothermal waters and steam, Iceland. Chemical Geology.

[CR48] Kharaka, Y. K., & Mariner, R.H. (1989). *Chemical geothermometers and their application to formation waters from sedimentary basins*. In: Naeser, N. D., & McCollin, T. H. (Eds.), Thermal history of sedimentary basins (pp 99–117). Springer-Verlag, New York.

[CR49] Kharaka YK, Lico MS, Law LM (1982). Chemical geothermometers applied to formation waters, Gulf of Mexico and California basins. American Association of Petroleum Geologists Bulletin.

[CR50] Langelier WF, Ludwig HF (1942). Graphical method for indicating the mineral character of natural waters. Jorunal of American Waterworks Association.

[CR51] Longinelli A, Selmo E (2003). Isotopic composition of precipitation in Italy: a first overall map. Journal of Hydrology.

[CR52] Martinelli G, Judd A (2004). Mud volcanoes of Italy. Geological Journal.

[CR53] Mattavelli L, Novelli L (1987). Geochemistry and habitat of natural gases in Italy. Organic Geochemistry.

[CR54] Minissale A, Vaselli O (2011). Karst springs as “natural” pluviometers: constraints on the isotopic composition of rainfall in the Apennines of central Italy. Applied Geochemistry.

[CR55] Minissale A, Magro G, Martinelli G, Vaselli O, Tassi F (2000). A fluid geochemical transect in the northern Apennines (central-northern Italy): fluid genesis and migration and tectonical implications. Tectonophysics.

[CR56] Molli G (2008). Northern Apennine-Corsica orogenic system: an update overview. Geological Society Special Publication.

[CR57] Montegrossi G, Tassi F, Vaselli O, Bidini E, Minissale A (2006). A new rapid and reliable method for the determination of reduced sulphur (S^2-^) species in natural water discharges. Applied Geochemistry.

[CR58] Mook WG, Bommerson JC, Staverman WH (1974). Carbon isotope fractionation between dissolved carbonate and gaseous carbon dioxide. Earth Planetary Science Letters.

[CR59] Mullis J (1988). Rapid subsidence and upthrusting in the Northern Apennines, deduced by fluid inclusion studies in quartz crystals from Porretta Terme. Schweizerische Mineralogische und Petrographische Mitteilungen.

[CR60] Parkhurst, D. L., & Appelo, C. A. J. (1999). User's guide to PHREEQC (version 2)—A computer program for speciation, batch-reaction, one-dimensional transport, and inverse geochemical calculations. *U.S. Geological Survey Water-Resources Investigations Report, 99–4259*, pp. 312.

[CR61] Picotti, V., & Pazzaglia, F. J. (2008). A new active tectonic model for the construction of the Northern Apennines mountain front near Bologna (Italy). *Journal of Geophysical Research, 113*, B08412.

[CR63] Pieri, M. (2001). Italian petroleum geology. In: Vai, G.B., & Martini, I. P. (Eds.), *Anatomy of an Orogen: The Apennines and Adjacent Mediterranean Basins (pp. 533–550)*. Dordrecht, Kluwer Academic Publisher.

[CR64] Principi G, Treves B (1984). Il sistema Corso-Appennino come prisma di accrezione. Riflessi sul problema generale del limite Alpi-Appennino. Memorie della Società Geologica Italiana.

[CR65] Ricci Lucchi, F. (1986). The Oligocene to recent foreland basins of the Northern Apennines. In: Allen, P. A. & Homewood, P. (Eds.), *Foreland basins (pp. 105–139)*, International Association of Sedimentologists Special Publication, 8.

[CR66] Roedder E, Bodnar RJ (1980). Geologic pressure determinations from fluid inclusion studies. Annual Review Earth and Planetary Sciences.

[CR67] Royden LH, Patacca E, Scandone P (1987). Segmentation and configuration of subducted lithosphere in Italy: an important control on thrust belt and foredeep basin evolution. Geology.

[CR68] Salata GG, Roelke LA, Cifuentes LA (2000). A rapid and precise method for measuring stable carbon isotope ratios of dissolved inorganic carbon. Marine Chemistry.

[CR69] Sano Y, Marty B (1995). Origin of carbon in fumarolic gas from island arcs. Chemical Geology.

[CR70] Schoell M (1980). The hydrogen and carbon isotopic composition of methane from natural gases of various origins. Geochimica et Cosmochimica Acta.

[CR71] Schoell M (1988). Multiple origins of methane in the Earth. Chemical Geology.

[CR72] Stauffer RE, Thompson JM (1984). Arsenic and antimony in geothermal waters of Yellowstone National Park, Wyoming, USA. Geochimica et Cosmochimica Acta.

[CR73] Tassi F, Bonini M, Montegrossi G, Capecchiacci F, Capaccioni B, Vaselli O (2012). Origin of light hydrocarbons in gases from mud volcanoes and CH_4_-rich emissions. Chemical Geology.

[CR74] Tassi F, Fiebig J, Vaselli O, Nocentini M (2012). Origins of methane discharging from volcanic-hydrothermal, geothermal and cold emissions in Italy. Chemical Geology.

[CR75] Vannucchi P, Remitti F, Bettelli G (2008). Geological record of fluid flow and seismogenesis along an erosive subducting plate boundary. Nature.

[CR76] Vaselli, O., Tassi, F., Montegrossi, G., Capaccioni, B., & Giannini, L. (2006). Sampling and analysis of fumarolic gases. *Acta Vulcanologica, 1–2*, 65–76.

[CR78] Vengosh A, Pankratov I (2005). Chloride/bromide and chloride/fluoride ratios of domestic sewage effluents and associated contaminated ground water. Ground Water.

[CR79] Wilhelm E, Battino R, Wilcock RJ (1977). Low-pressure solubility of gases in liquid water. Chemical Review.

[CR80] Ventura B, Pini GA, Zuffa GG (2001). Thermal history and exhumation of the Northern Apennines (Italy): evidences from combined apatite fission track and vitrinite reflectance data from foreland basin sediments. Basin Research.

[CR81] Whiticar MJ (1999). Carbon and hydrogen isotope systematic of bacterial formation and oxidation of methane. Chemical Geology.

[CR82] WHO (2011). Guidelines for drinking-water quality.

[CR83] Wilkinson JJ, Stoffell B, Wilkinson CC, Jeffries TE, Appold MS (2009). Anomalously metal-rich fluids form hydrothermal ore deposits. Science.

[CR84] Zattin, M. (1998). *Apatite thermochronology of the Marnoso-arenacea succession (Miocene, Northern Apennines)* (pp. 1–139). PhD Thesis, University of Bologna.

[CR85] Zhang J, Quay PD, Wilbur DO (1995). Carbon isotope fractionation during gas-water exchange and dissolution of CO_2_. Geochimica et Cosmochimica Acta.

